# A complete twelve-gene deletion null mutant reveals that cyclic di-GMP is a global regulator of phase-transition and host colonization in *Erwinia amylovora*

**DOI:** 10.1371/journal.ppat.1010737

**Published:** 2022-08-01

**Authors:** Roshni R. Kharadi, Kayla Selbmann, George W. Sundin

**Affiliations:** Department of Plant, Soil and Microbial Sciences, Michigan State University, East Lansing, Michigan, United States of America; University of Cambridge, UNITED KINGDOM

## Abstract

Cyclic-di-GMP (c-di-GMP) is an essential bacterial second messenger that regulates biofilm formation and pathogenicity. To study the global regulatory effect of individual components of the c-di-GMP metabolic system, we deleted all 12 diguanylate cyclase (*dgc*) and phosphodiesterase (*pde*)-encoding genes in *E*. *amylovora* Ea1189 (Ea1189Δ12). Ea1189Δ12 was impaired in surface attachment due to a transcriptional dysregulation of the type IV pilus and the flagellar filament. A transcriptomic analysis of surface-exposed WT Ea1189 and Ea1189Δ12 cells indicated that genes involved in metabolism, appendage generation and global transcriptional/post-transcriptional regulation were differentially regulated in Ea1189Δ12. Biofilm formation was regulated by all 5 Dgcs, whereas type III secretion and disease development were differentially regulated by specific Dgcs. A comparative transcriptomic analysis of Ea1189Δ8 (lacks all five enzymatically active *dgc* and 3 *pde* genes) against Ea1189Δ8 expressing specific *dgcs*, revealed the presence of a dual modality of spatial and global regulatory frameworks in the c-di-GMP signaling network.

## Author summary

Cyclic-di-GMP dependent regulation, in the context of biofilm formation, has been studied in several bacterial systems. However, there are fewer studies exploring the role of individual genetic components related to cyclic-di-GMP, given the presence of an often large number of diguanylate cyclase and phosphodiesterase enzymes in bacterial systems. To explore the evolutionary dependencies related to cyclic-di-GMP in *E*. *amylovora*, we used a collective elimination approach, whereby all of the enzymes involved in cyclic-di-GMP metabolism were eliminated from the system. This approach enabled us to highlight the importance of cyclic-di-GMP in plant xylem colonization due to its effect on surface attachment. Additionally, we highlight the differential regulatory impact of each of the *dgc*/*pde* genes on critical virulence factors in *E*. *amylovora* with a genetic framework that is not affected by any enzymatic redundancy or antagonism. We further use a transcriptomic approach to reveal evidence of regulatory localization/heterogeneity mediated by each of the *dgc* genes.

## Introduction

The bis (3’,5’)-cyclic diguanosine monophosphate (c-di-GMP) signaling system is a ubiquitous and effective adaptation by which bacteria can gather sensory input from environmental stimuli and correspondingly regulate cellular function to then effectively employ the most appropriate response for survival within a particular environment or for host colonization in a pathogenic context [1,2]. Bacterial c-di-GMP networks consist of diguanylate cyclase (Dgc) enzymes, marked by the presence of a GGDEF motif, that synthesize c-di-GMP from two molecules of guanosine tri-phosphate (GTP) substrate, and phosphodiesterase (Pde) enzymes, containing an EAL and/or HD-GYP domain that c-di-GMP into pGpG (5’-phosphoguanylyl-(3’➔5’)-guanosine). The HD-GYP class of Pdes and oligoribonucleases can directly hydrolyze pGpG into guanosine mono-phosphate (GMP) subunits. In *E*. *amylovora*, five active Dgcs (EdcA-E) and three active Pdes (PdeA-C) [EAL domain containing proteins] have been functionally characterized [3,4]. As documented in other bacterial systems including *Escherichia coli* (29 Dgcs/Pdes), *Pseudomonas aeruginosa* (38 Dgcs/Pdes), *Salmonella enterica* [5,6] (20 Dgcs/Pdes), and *Vibrio cholerae* [7,8] (61 Dgcs/Pdes), the presence of a large number of Dgcs/Pdes and systemic functional redundancy is often observed. The retention of such a high number of Dgcs and Pdes in bacterial pathogen systems raises questions about the evolutionary significance of developing multilayered genetic control strategies.

The primary approach used to study the regulatory effect of individual components of c-di-GMP turnover is by deleting one or more genes in combination and evaluating phenotypic and regulatory changes. In *Salmonella*, Solano et al. [5] generated a multigene mutant that eliminated 12 *dgc* genes and Sarenko et al. [9], studied a collective group of single deletion mutants of each of the 29 individual Dgc and Pde encoding genes in *Escherichia coli*. Both studies indicated that the functional effect of specific Dgcs and Pdes on virulence phenotypes could be either dependent or independent of their metabolic activity towards c-di-GMP. Abel et al. [10] generated a c-di-GMP null strain by deleting the active Dgc and Pde encoding genes in *Caulobacter crescentus* and demonstrated the impact on growth, motility and surface attachment. While the targeted elimination of one or more genes in the c-di-GMP regulatory system is a straightforward approach that can highlight significant changes in the regulation of critical virulence factors, the presence of multiple other enzymes can, through redundancy or antagonistic enzymatic effect, mask some of the effects occurring due to the loss of one particular gene and thus the resulting downstream regulatory effect. Additionally, the overall regulatory effect of each of these enzyme classes has not been explored in a background that does not include any additional impedance/interaction with any of the other components in the network.

In order to address these concerns and with the overarching aim of exploring the role of the global effect of c-di-GMP on virulence manifestation in the host, we used a c-di-GMP systematic deletion approach in *Erwinia amylovora*, the causal agent of fire blight disease of rosaceous plants [11]. An evolutionary adaptation that helps *E*. *amylovora* systemically colonize the apple host is its ability to attach to the walls of xylem vasculature and form robust biofilms within the xylem channels, thus enabling extensive proliferation of the pathogen during this stage of the disease cycle [12]. Cyclic-di-GMP is one of the critical factors that regulates biofilm formation in *E*. *amylovora* [3,4], and elevated intracellular levels of c-di-GMP have been correlated with increased levels of biofilm development in static and flow-based *in vitro* systems [4,13]. In addition to biofilm formation, c-di-GMP also negatively regulates type III secretion system (T3SS) mediated virulence via the transcriptional downregulation of *hrpL* (alternate sigma factor required for the transcription of *hrp* genes) and a reduction in the amount of the T3SS effector DspE (pathogenicity factor) that is transferred into host cells [3,4].

We hypothesized that the retention of multiple Edc and Pde enzymes in this system was due to adaptive functional divergence. To test our hypothesis, we eliminated all 12 genes of the c-di-GMP metabolic network in *E*. *amylovora* Ea1189 (Ea1189Δ12), resulting in an *E*. *amylovora* strain with no background c-di-GMP formative, degradative, or signaling activity. We then examined the impact of specific Edcs on several virulence factors. A transcriptomic approach was also used to examine the global impact of c-di-GMP during biofilm initiation and to study the regulatory network mediated by specific Edcs.

## Results

### *E. amylovora* encodes an array of 12 proteins with GGDEF and/or EAL motifs

**Fig 1:**
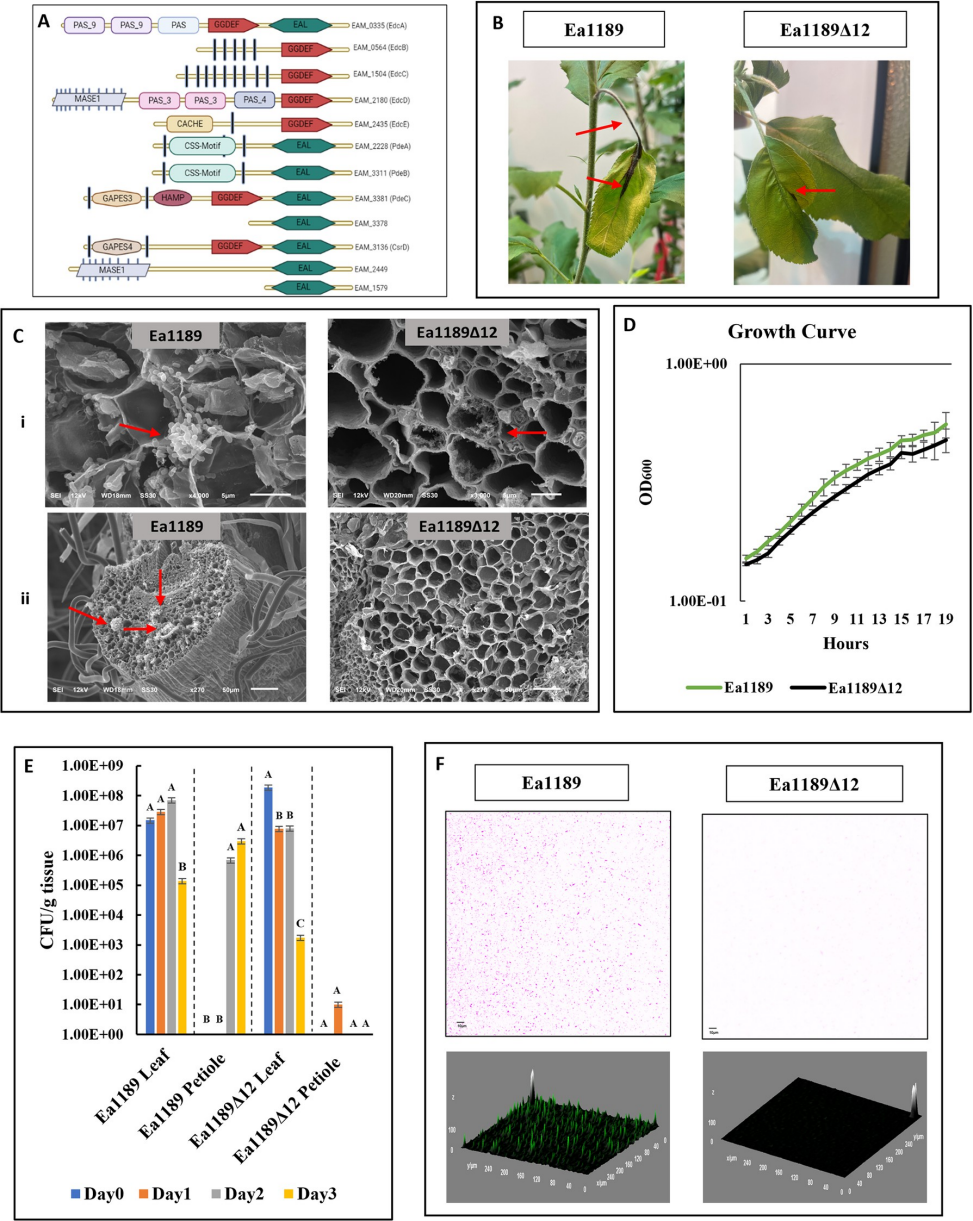
C-di-GMP is essential for host colonization. A) A representative protein domain architectural overview of all identified proteins in *E*. *amylovora* Ea1189 that contain a GGDEF and/or EAL domain. Filled black vertical bars represent transmembrane helices on the N-terminal domain. The image was created using Biorender. B) Images depicting disease progression in apple shoots infected with Ea189 and Ea1189Δ12 at 3dpi. While Ea1189 infected shoots show signs of infection the leaf and the petiole (red arrows), shoots infected with Ea1189Δ12 show minor signs of necrosis limited to the apoplast region in the leaf (red arrow) C) Scanning electron micrographs depicting sectional images of the i) apoplast and ii) xylem tissue of young apple shoot tips 3 dpi with *E*. *amylovora* Ea1189 and Ea1189Δ12. Widespread colonization was observed when shoot tips were inoculated with WT Ea1189 in both the apoplast and the xylem. However, shoots inoculated with Ea1189Δ12 showed less severe bacterial colonization in the apoplast and no evidence of any biofilm development within the xylem tissue in the petiole. D) Growth patterns *in vitro* for Ea1189 and Ea1189Δ12 don’t show any significant difference. E) Bacterial population counts over the course of 3 days post inoculation of young shoot tips with WT Ea1189 and Ea1189Δ12. Leaf and petiole samples were separately examined. Over a time span of 72 hrs, the bacterial population of Ea1189 increases within the petiole and declines within the apoplast, whereas populations of Ea1189Δ12 decline in the leaf tissue and are at undetectable levels in the petiole. Error bars represent standard errors of the means. Tukey’s HSD (honestly significant difference) (*P* < 0.05) test was used to determine statistical significance over the course of the experiment for each tissue type. F) Z-stacked confocal microscopy images (color inverted) showing the overall attachment occurring within the flow chamber one hour after the introduction of either Ea1189 or Ea1189Δ12 cells into the chamber, followed by the flushing of the chamber with 0.5X PBS. Ea1189 cells displayed widespread even attachment with interspersed patches of elevated fluorescence signal indicating potential multilayered attachment. Ea1189Δ12 cells failed to attach to the chamber surface.

The regulatory impact of the *edc* and *pde* genes on virulence has been studied separately in *E*. *amylovora* through the assessment of phenotypic variation in mutants lacking one or more of these genes [3,4]. In this study, we aimed to decipher the evolutionary role of both the formative and degradative components of the overall c-di-GMP system. In order to do so, we generated a systemic null mutant lacking all 12 genes that encode proteins containing a GGDEF and/or EAL motif (as annotated by Pfam v 33.1) [14]. Proteins containing HD-GYP motifs are absent in *E*. *amylovora* [4]. The list of 12 proteins along with their domain architecture is graphically mapped in Fig 1A. EdcA-E have been previously mapped and characterized by Edmunds et al. [3]. The functional traits regulated by PdeA-C have been studied by Kharadi et al. [4]. EAM_3378, EAM_3136 (CsrD) [15], EAM_2449, and EAM_1579 encode proteins with degenerate GGDEF/EAL domains as designated on Pfam [14]. The N-terminal end of all proteins except EdcA, EAM_3378 and EAM_1579 includes a wide array of periplasmic sensory domains and multiple transmembrane helices (as predicted by TMHMM Server v 2.0) [16]. EdcA, PdeC and CsrD contain both GGDEF and EAL domains (Fig 1A).

### C-di-GMP is essential for systemic host colonization

To determine the impact of a complete c-di-GMP null mutant on virulence in planta, young apple leaves at the tips of branches were inoculated with *E*. *amylovora* Ea1189 or Ea1189Δ12 and were tracked for disease progression. Apple branches infected with Ea1189Δ12 showed a significantly-reduced external manifestation of necrotic tissue development, and that such necrosis was limited to apoplastic leaf regions (Fig 1B). Using scanning electron microscopy (SEM), we also checked for any patterns of bacterial proliferation and localization within the apoplast and xylem vessels. *E*. *amylovora* Ea1189 cells were detected abundantly in the apoplast 72 hpi (hours post inoculation). In the xylem vessels, Ea1189 cells were able to attach and develop extensive biofilms with an abundance of EPS, thus, functionally impeding the channels (Fig 1C (i and ii)). In contrast, the apoplast region of leaves infected with Ea1189Δ12 showed relatively few pockets of cells with some extracellular material, whereas the xylem vessels showed no microscopic signs of colonization despite the appearance of some minor necrotic lesions on the leaf surface near the site of inoculation and no cellular attachment, exopolysaccharide (EPS) generation, or biofilm formation was detected (Fig 1C (i and ii)). There were no apparent differences in the growth patterns for Ea1189 and Ea1189Δ12 *in vitro* (Fig 1D). Bacterial population counts taken separately from leaf and petiole tissue of shoots infected with Ea1189 and Ea1189Δ12 indicated that 24 hpi, a majority of the detectable bacterial population was still restricted to the leaf tissue and was detected in shoots infected with both strains at levels similar to ones at the time of inoculation (between 10^7^and 10^8^ CFU/g of tissue) (Fig 1E). At 48 hpi, shoots infected with Ea1189 contained detectable levels of bacterial populations in the petiole at ~10^6^ CFU/g, which then increased slightly at 72 hpi and inversely, the bacterial load decreased in the leaf tissue at 72 hpi (Fig 1E). Within the leaf tissue, Ea1189Δ12 populations followed a similar trend and declined significantly by 72 hpi; however, apart from a minor level of bacteria detected in the apoplast at 24 hpi, there was no detectable bacterial population present in the apoplast during the course of the experiment (Fig 1E).

### Surface attachment is a limiting factor for biofilm development and is dependent on c-di-GMP signaling in *E. amylovora*

**Fig 2:**
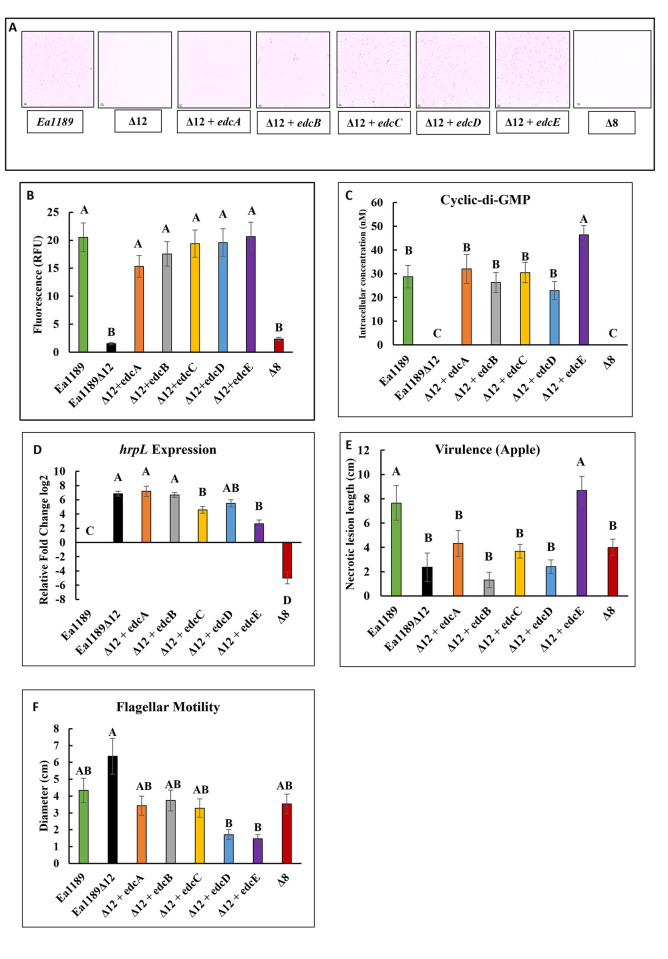
Edcs differentially regulate biofilm formation and virulence. A) Confocal images (color inverted) and B) relative GFP intensity of flow cells inoculated with GFP labelled WT Ea1189, Ea1189Δ8 Ea1189Δ12 and Ea1189Δ12 complemented with individual *edc* genes. Bacterial inoculum was introduced into the flow cells and allowed to incubate for 1 h before being flushed out, followed by incubation under flow for 5 h. The flow cells were then imaged along a z-plane to assess the volume of bacterial adhesion to within the chamber. Ea1189Δ12 and Ea1189Δ8 are impaired in biofilm formation relative to Ea1189. Complementation of Ea1189Δ12 with the individual *edc* genes restores the biofilm formation to levels similar to Ea1189. C) C-di-GMP formation was attenuated in Ea1189Δ8 and Ea1189Δ12, and the complementation of Ea1189Δ12 with *edcA-E* was able to individually restore c-di-GMP levels to WT Ea1189 levels with the highest increase recorded in Ea1189Δ12/*edcE*. D) *hrpL* transcript levels, relative to WT Ea1189 were significantly increased in Ea1189Δ12, and complementation with *edcC* and *edcE* was able to significantly reduce the transcript levels as compared to Ea1189Δ12. Ea1189Δ8 had significantly lower hrpL transcript levels compared to Ea1189 and Ea1189Δ12. E) Virulence in apple shoots was significantly reduced in Ea1189Δ8 and Ea1189Δ12 relative to Ea1189. Only complementation with *edcE* was able to restore WT levels of shoot blight in Ea1189Δ12. F) Flagellar motility was not significantly affected in Ea1189Δ12/ Ea1189Δ8 compared to WT Ea1189. Complementation of Ea1189Δ12 with *edcD* and *edcE* was able to significantly reduce motility as compared to Ea1189Δ12. Error bars represent standard errors of the means. Tukey’s HSD (honestly significant difference) (*P* < 0.05) test was used to determine statistical significance for all experiments.

Since an initial *in vitro* assessment indicated a lack of biofilm formation in Ea1189Δ12 compared to WT Ea1189 (further discussed and presented in Fig 2), we analyzed if this was due to an impairment in surface attachment, which could be limiting further biofilm development. We monitored the interaction of GFP labelled cells with the base of the flow chamber upon initial contact using TIRF (total internal reflection fluorescence) microscopy. S1 and S2 Videos present collated images in the form of a time lapse video presenting the dynamics of surface interaction of Ea1189 (S1 Video) and Ea1189Δ12 (S2 Video) cells introduced into the chamber. Ea1189 cells approached the basal portion of the flow cell, and a subset of them would attach in every frame of the video (S1 and S2 Videos). Over time, this led to a saturation of the image frame with GFP signal from attached cells. In contrast, several Ea1189Δ12 cells approached the basal surface but failed to attach irreversibly to the surface. Due to the pace of the video, this occurrence plays out in the form of momentary GFP signal increases and lapses as cells approach and then reproach from the surface (S1 and S2 Videos). An assessment of the flow chamber at the end of this experiment by flushing out the planktonic cells revealed that Ea1189 cells were able to evenly attach to the base of the flow chamber, and Ea1189Δ12 were unable to do so (Fig 1F).

### Diguanylate cyclases differentially contribute to biofilm formation and virulence in *E. amylovora*

Through the restoration of individual *edc* genes (*edcA-E*) in Ea1189Δ12, cells were able to regain the ability to attach to a surface and form biofilms *in vitro* (Fig 2A). Previous studies, and the c-di-GMP quantitative data from this study, indicated that there were five enzymatically active diguanylate cyclases (EdcA-E) and three phosphodiesterases (PdeA-C) that could quantitatively impact the intracellular levels of c-di-GMP in *E*. *amylovora* [3,4]. Further, since proteins with degenerate EAL motifs have been associated with c-di-GMP binding and some downstream regulation, we decided to retain the four other genes in our study (CsrD, EAM_3378, EAM_2449 and EAM_1579) so as to not disrupt any such potential signaling activity [17,18]. Thus, we eliminated the eight genes encoding for these enzymes (*edcA-E* and *pdeA-C*), and generated Ea1189Δ8 which was also severely reduced in biofilm formation as quantified within flow cells (Fig 2A and 2B). All five Edcs contributed to attachment, and the restoration of even a single *edc* gene could restore WT levels of overall biofilm formation in a flow-based system (Fig 2A and 2B). Ea1189Δ12 complemented with *edcA-D* also generated WT Ea1189 levels of c-di-GMP *in vitro*, with Ea1189Δ12/*edcE* generating significantly higher intracellular c-di-GMP levels, relative to Ea1189 (Fig 2C). No c-di-GMP was detected in Ea1189Δ8 (Fig 2C).

*hrpL* encodes an alternate sigma factor that regulates the transcription of T3SS related genes in *E*. *amylovora* [19]. *hrpL* transcript levels *in vitro* were significantly elevated in Ea1189Δ12 relative to Ea1189, whereas the transcript levels were significantly reduced in Ea1189Δ8 relative to Ea1189 (Fig 2D). Ea1189Δ12 complemented with *edcC* or *edcE* exhibited significantly-reduced *hrpL* transcript levels relative to the other complemented strains, however all complemented strains exhibited *hrpL* transcript levels greater that Ea1189 (Fig 2D). Virulence in apple shoots was significantly reduced in Ea1189Δ12 and Ea1189Δ8 relative to Ea1189, and complementation of Ea1189Δ12 with *edcE* was able to elevate virulence and restore WT levels of shoot blight (Fig 2E). Flagellar motility was not significantly different in both Ea1189Δ12 and Ea1189Δ8 relative to Ea1189 (Fig 2F). The complementation of Ea1189Δ12 with *edcD* and *edcE* resulted in significantly reduce levels of motility in the complemented strains as compared to Ea1189Δ12 (Fig 2F).

### The type IV pilus in conjunction with the flagellar filament mediates surface attachment in a c-di-GMP dependent manner in *E. amylovora*

**Fig 3:**
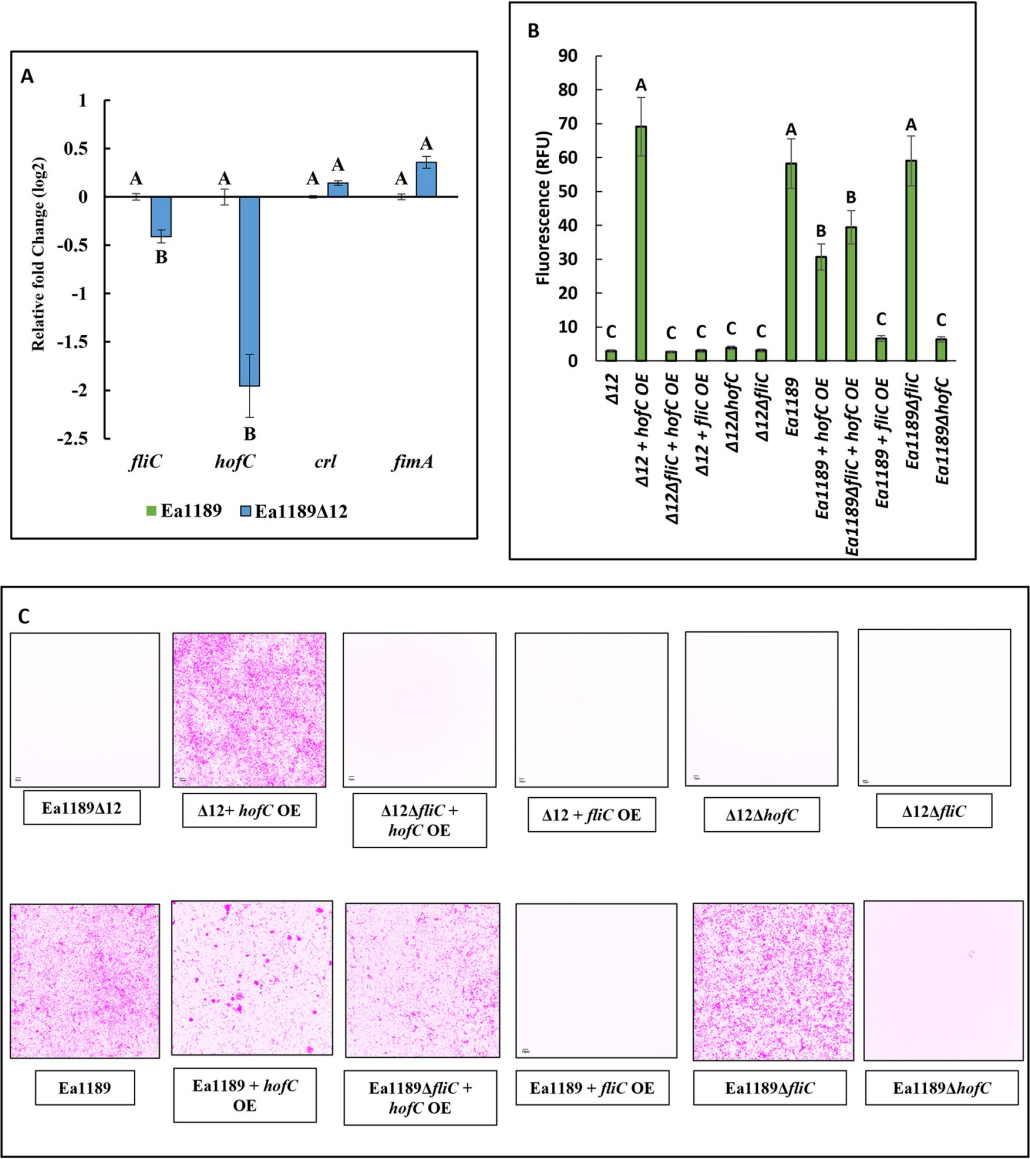
Type IV pilus and the flagellum mediate surface attachment. A) Transcript levels of *fliC* (flagellar filament) and *hofC* (type IV pilus assembly platform protein), were significantly reduced in Ea1189Δ12 relative to WT Ea1189. *crl* (curli fimbriae activator) and *fimA* (fimbrial subunit) transcript levels were not significantly different among the two strains. B) Relative bacterial adhesion GFP intensity representing the level and C) Confocal z-stacked images (color inverted) of attachment within flow cells 1hr after incubation with Ea1189 and Ea1189Δ12 lacking or overexpressing *fliC* and/or *hofC*. The overexpression of *hofC* could restore attachment in Ea1189Δ12 to WT Ea1189 levels, however, this impact was lost if the overexpression occurred in the absence of *fliC*. WT Ea1189 showed considerable levels of cellular surface attachment, which diminished upon the overexpression of *fliC* and the deletion of *hofC*. The deletion of *fliC* and/or the overexpression of *hofC* did alter but did not abolish attachment in Ea1189. Error bars represent standard errors of the means. Tukey’s HSD (honestly significant.

In order to investigate if the systematic deletion of c-di-GMP affected any particular extracellular appendages that could contribute to the lack of surface attachment, we measured the relative transcript abundance of four genes representative of different appendages, including *fliC* (flagellar filament), *hofC* (type IV pilus assembly platform protein), *crl* (curli fimbriae activator) and *fimA* (fimbrial subunit) [20–24]. Surface-exposed Ea1189Δ12 cells showed a significant decline in the transcript abundance of *fliC* and *hofC*, whereas levels of *fimA* were increased and *crl* unchanged relative to WT Ea1189 (Fig 3A). Induced overexpression of *fliC* in Ea1189Δ12 did not result in any visible changes in the level of attachment in flow cells (Fig 3B and 3C). However, the overexpression of *hofC* significantly elevated attachment in Ea1189Δ12, and restored attachment to the level of the WT Ea1189. Indicative of a co-dependence of the type IV pilus on the flagellum, when *hofC* was overexpressed in Ea1189Δ12Δ*fliC*, the level of surface attachment did not increase (Fig 3B and 3C). Ea1189Δ12Δ*fliC* and Ea1189Δ12Δ*hofC* also showed no detectable signs of attachment within flow cells (Fig 3B and 3C). In WT Ea1189, relative to Ea1189Δ12, the level of cellular attachment within flow cells was significantly higher. The deletion of *fliC* in Ea1189, and the overexpression of *hofC* in both Ea1189 and in Ea1189Δ*fliC* allowed for the retention of cellular attachment to the flow cell surface, despite a quantitative decline in terms of RFU detection relative to Ea1189 when *hofC* was overexpressed (Fig 3B and 3C). Attachment to the flow chamber was significantly reduced in Ea1189Δ*hofC* and when *fliC* was overexpressed in Ea1189 (Fig 3B and 3C).

### C-di-GMP regulates the transcription of several critical targets during biofilm initiation

**Fig 4:**
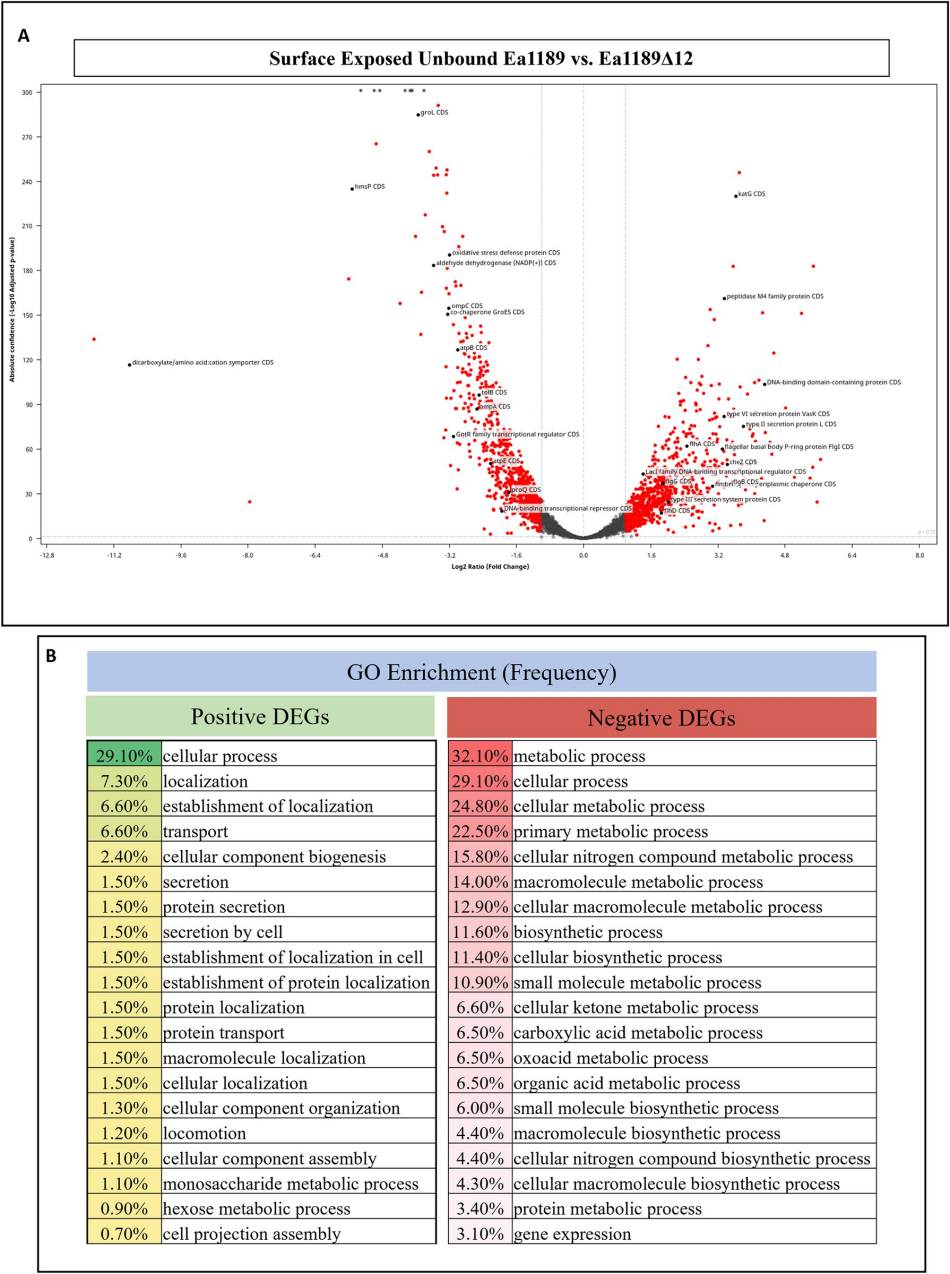
Global c-di-GMP dependent regulation during biofilm initiation. A) A volcano plot highlighting critical differentially expressed genes (DEGs) within surface exposed WT Ea1189 vs. Ea1189Δ12 cells analyzed via RNA-seq analysis. A DESeq2 FDR cutoff p-value of 0.05 was used and all DEGs highlighted in red have a two fold change (log2) in expression. The comparison revealed a total of 320 positively and 235 negatively expressed DEGs with functions including metabolism, extracellular appendage regulation and overall transcriptional/post-transcriptional regulators. B) GO enrichment analysis showing the top 20 overrepresented categories for the positive and negative DEGs, along with the overall frequency of gene/target occurrence within the DEG list.

To evaluate the global transcriptional impact of the presence or absence of c-di-GMP during biofilm initiation, we compared the transcriptome of WT Ea1189 and Ea1189Δ12 through an RNAseq assessment of cells harvested from flow chambers after 1 hour of exposure. Overall, we detected a total of 320 positively-affected and 235 negatively-affected differentially expressed genes (DEGs) in Ea1189Δ12 relative to WT Ea1189, based on a DESeq2 FDR cutoff of 0.05 and a fold change of two (log2) (Fig 4A). Gene ontology (GO) enrichment analysis for over-representation in biological function categories indicated that the top 20 categories among the positive DEGs comprised heavily of protein transport, localization and secretion along with cellular movement, whereas the negative DEGs mainly comprised of metabolic and biosynthetic genes (Fig 4B). A complete list of DEGs is provided in supplemental datasheet A. Among the top negatively regulated genes were multiple protease activity related targets including EAM_RS16610 (insulinase encoding gene), *htpX*, *yccA* and *hslU*. Several metabolic genes including *rbsD*, *acs*, *pckA*, *gapA*, *ribB* and EAM_RS12095 (NADP encoding gene) were also in the negatively expressed category. The other major categories of negatively expressed genes were those of protein folding/transport and regulatory genes including *groL*, *mglB*, *pspB*, *hmsP* and EAM_RS05285 (encodes for a leucine-rich repeat domain containing protein). The top 25 negative DEGs and their relevant characteristics including statistical data are listed in Table 1. Among the positively expressed genes were several appendage/transport/secretion system related genes including *fimA*, *sctD*, *tssJ*, *sctV*, *yeeU*, *tssL* and *cbtA*. Metabolic genes including *glgB*, *glgX*, *galB*, *glgC* were also among the positive DEGs. The top 25 positive DEGs and their relevant characteristics including statistical data are listed in Table 2. q-RT-PCR was used to validate gene expression data from the RNAseq experiment using a subset of genes (S1A Fig).

**Table 1:** A list of the 25 most negatively regulated genes in Ea1189Δ12 relative to WT Ea1189.

Locus Tag	Gene ID	Product	Differential Expression Log2 Ratio	Differential Expression p-value
EAM_RS16610	insulinase family protein CDS	insulinase family protein	-11.66137022	1.9342E-136
EAM_RS16615	dicarboxylate/amino acid:cation symporter CDS	dicarboxylate/amino acid:cation symporter	-10.81381354	5.2721E-119
EAM_RS19445	hypothetical protein CDS	hypothetical protein	-7.950021219	9.05238E-26
EAM_RS11745	DUF1471 domain-containing protein CDS	DUF1471 domain-containing protein	-5.590416774	3.0468E-177
EAM_RS16620	*hmsP* CDS	biofilm formation regulator HmsP	-5.50909201	4.6447E-238
EAM_RS05505	amino acid ABC transporter substrate-binding protein CDS	amino acid ABC transporter substrate-binding protein	-5.30482975	0
EAM_RS05285	leucine-rich repeat domain-containing protein CDS	leucine-rich repeat domain-containing protein	-4.983851837	0
EAM_RS12130	APC family permease CDS	APC family permease	-4.936317974	1.0207E-268
EAM_RS00070	*rbsD* CDS	D-ribose pyranase	-4.850327937	0
EAM_RS09500	*htpX* CDS	protease HtpX	-4.366262025	1.1091E-160
EAM_RS10695	*mglB* CDS	galactose/glucose ABC transporter substrate-binding protein MglB	-4.247135295	0
EAM_RS09990	hypothetical protein CDS	hypothetical protein	-4.127207088	0
EAM_RS06765	*yccA* CDS	FtsH protease modulator YccA	-4.094370008	0
EAM_RS02625	glucitol/sorbitol permease IIC component CDS	glucitol/sorbitol permease IIC component	-4.082619297	0
EAM_RS01695	*acs* CDS	acetate—CoA ligase	-3.99767443	4.442E-206
EAM_RS02150	*groL* CDS	chaperonin GroEL	-3.934347101	3.1088E-288
EAM_RS12125	dihydrodipicolinate synthase family protein CDS	dihydrodipicolinate synthase family protein	-3.872046115	9.868E-140
EAM_RS07820	*spy* CDS	ATP-independent periplasmic protein-refolding chaperone Spy	-3.854421997	2.9565E-168
EAM_RS15970	*pckA* CDS	phosphoenolpyruvate carboxykinase (ATP)	-3.798261114	0
EAM_RS09315	*gapA* CDS	glyceraldehyde-3-phosphate dehydrogenase	-3.766942859	1.4129E-220
EAM_RS12115	4-hydroxyproline epimerase CDS	4-hydroxyproline epimerase	-3.671073648	1.6735E-263
EAM_RS12095	aldehyde dehydrogenase (NADP(+)) CDS	aldehyde dehydrogenase (NADP(+))	-3.568133834	2.0155E-186
EAM_RS08865	*pspB* CDS	envelope stress response membrane protein PspB	-3.567808565	2.2995E-247
EAM_RS14660	*ribB* CDS	3,4-dihydroxy-2-butanone-4-phosphate synthase	-3.507981226	2.2124E-252
EAM_RS00630	*hslU* CDS	HslU—HslV peptidase ATPase subunit	-3.473822217	1.301E-247

**Table 2:** A list of the 25 most positively regulated genes in Ea1189Δ12 relative to WT Ea1189.

Locus Tag	Gene ID	Product	Differential Expression Log2 Ratio	Differential Expression p-value
EAM_RS14005	TIGR03756 family integrating conjugative elementprotein CDS	TIGR03756 family integrating conjugative elementprotein	5.653778414	1.02E-54
EAM_RS14820	AlpA family phage regulatory protein CDS	AlpA family phage regulatory protein	5.570644235	1.36E-25
EAM_RS16075	*glgB* CDS	1,4-alpha-glucan branching enzyme	5.483356916	7.21E-186
EAM_RS01250	fimbrial protein A precursor CDS	fimbrial protein A precursor	5.467190074	2.17E-49
EAM_RS16430	hypothetical protein CDS	hypothetical protein	5.404620393	5.29E-42
EAM_RS19320	*glgX* CDS	glycogen debranching protein GlgX	5.201160707	5.60E-154
EAM_RS03470	hypothetical protein CDS	hypothetical protein	5.029951978	1.09E-42
EAM_RS14000	integrating conjugative element protein CDS	integrating conjugative element protein	4.820222392	1.09E-89
EAM_RS06020	YIP1 family protein CDS	YIP1 family protein	4.539960781	4.03E-127
EAM_RS09185	*galB* CDS	4-oxalmesaconate hydratase	4.49334628	2.78E-58
EAM_RS04295	amino acid ABC transporter permease CDS	amino acid ABC transporter permease	4.448825708	2.15E-66
EAM_RS14195	*sctD* CDS	type III secretion system inner membrane ring subunit SctD	4.42440179	4.41E-64
EAM_RS04290	transporter substrate-binding domain-containing protein CDS	transporter substrate-binding domain-containing protein	4.339006646	5.35E-73
EAM_RS13995	DNA-binding domain-containing protein CDS	DNA-binding domain-containing protein	4.322676558	9.56E-106
EAM_RS02010	*tssJ* CDS	type VI secretion system lipoprotein TssJ	4.310343973	6.61E-13
EAM_RS19315	*glgC* CDS	glucose-1-phosphate adenylyltransferase	4.272326359	2.41E-154
EAM_RS03515	prepilin-type N-terminal cleavage/methylation domain-containing protein CDS	prepilin-type N-terminal cleavage/methylation domain-containing protein	4.248823084	1.03E-41
EAM_RS12610	chemotaxis response regulator protein-glutamate methylesterase CDS	chemotaxis response regulator protein-glutamate methylesterase	4.187095164	9.64E-78
EAM_RS14190	*sctV* CDS	type III secretion system export apparatus subunit SctV	4.186154654	1.22E-108
EAM_RS14010	TIGR03757 family integrating conjugative elementprotein CDS	TIGR03757 family integrating conjugative elementprotein	4.13782143	7.78E-46
EAM_RS01905	*tssK* CDS	type VI secretion system baseplate subunit TssK	4.109363563	6.27E-87
EAM_RS09200	NAD(P)-dependent oxidoreductase CDS	NAD(P)-dependent oxidoreductase	4.102789474	4.89E-67
EAM_RS14800	type IV toxin-antitoxin system YeeU family antitoxin CDS	type IV toxin-antitoxin system YeeU family antitoxin	4.10085975	2.44E-37
EAM_RS01910	type VI secretion system protein TssL, short form CDS	type VI secretion system protein TssL, short form	4.092459487	1.93E-53
EAM_RS14795	TA system toxin CbtA family protein CDS	TA system toxin CbtA family protein	4.085573729	2.78E-31

### Regulatory divergence among the diguanylate cyclases of *E. amylovora*

Some studies have presented evidence of c-di-GMP/ downstream signaling localization in specific regions of the cell during different stages of the cell cycle [25–28]. Thus, we wanted to determine, through a transcriptomic approach, if the overarching pattern of regulatory targets affected by the c-di-GMP generated by each individual Edc enzyme was that of overlap or of divergence. A phylogenetic analysis using a compilation of the top 500 pblast search results (p-value cutoff of 0.05 using a BLOSUM62 matrix with conditional compositional score adjustment) for of all 5 Edcs indicated no evidence of relatively recent acquisition from non-Erwiniaceae species (Sup. datasheet B). This suggested the possibility of functional divergence occurring due to prolonged recurring evolutionary pressure imposed on the genes [29–31].

Initial phenotypic assessments conducted on Ea1189Δ8 indicated that in terms of c-di-GMP production, shoot blight and flagellar motility, Ea1189Δ8 was not significantly different from Ea1189Δ12 (Fig 2A, 2B, 2C, 2E and 2F). However, in terms of *hrpL* transcription, Ea1189Δ8 showed contrasting trends relative to Ea1189Δ12 with a significant reduction in *hrpL* transcription relative to WT Ea1189 (Fig 2D). Since any manner of exogenously generated c-di-GMP in Ea1189Δ8 would likely not be hydrolyzed within the cell, we sought to further enrich for this regulatory impact by the induced overexpression of each of the five *edc* genes in Ea1189Δ8 and then study the DEG patterns through RNAseq.

**Fig 5:**
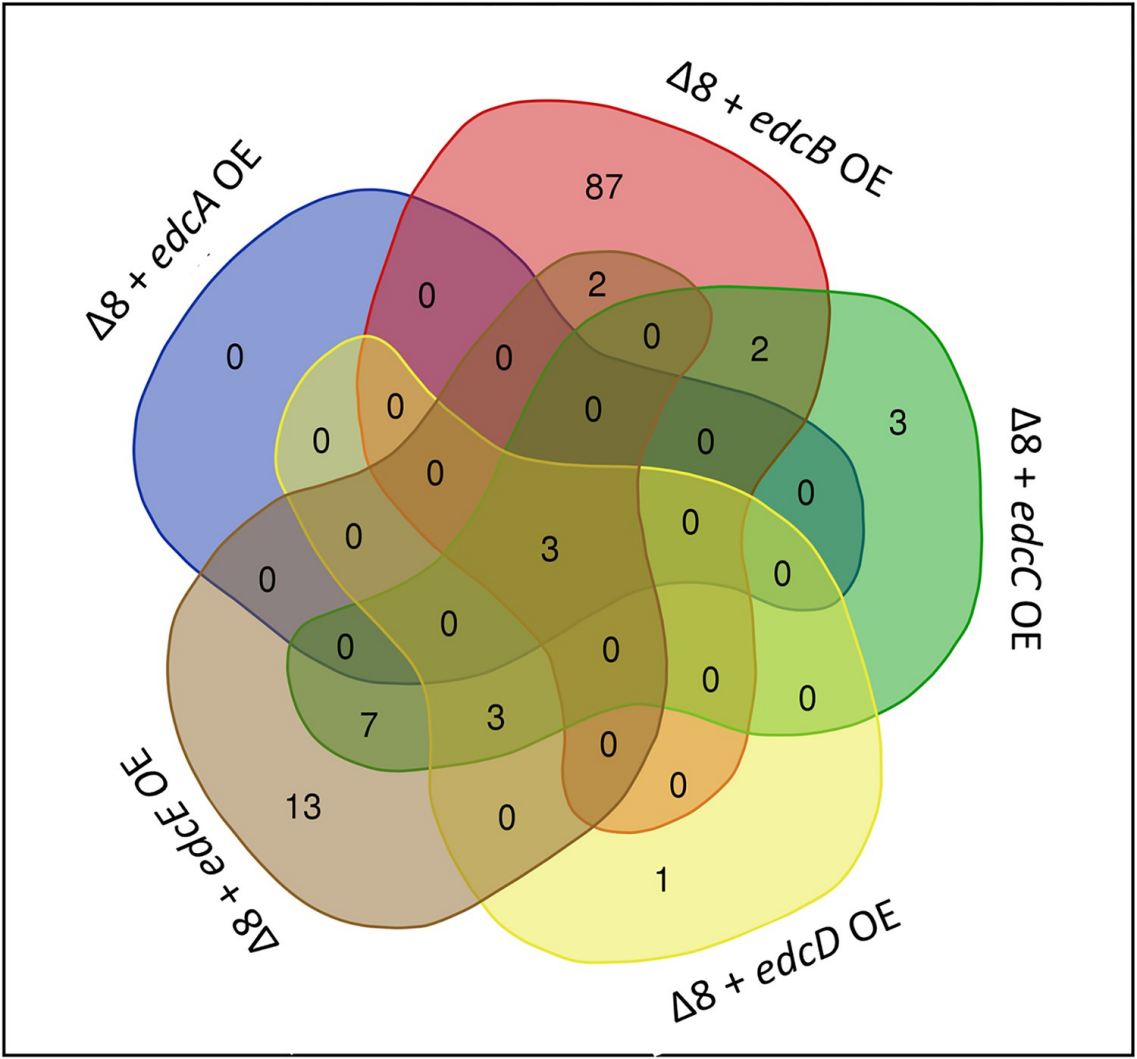
Regulatory divergence among the Edcs. A) A venn diagram representing the distribution of the DEGs in Ea1189Δ8 strain overexpressing individual *edc* genes measured via RNAseq. A total of 121 DEGs both positively and negatively affected in expression were found after being filtered through a DESeq2 FDR cutoff of 0.05 with at least a two fold change (log2) individually for each comparative condition of Ea1189Δ8 vs Ea1189Δ8 overexpressing an individual *edc* gene. Ea1189Δ8 overexpressing *edcB* had the highest number of uniquely regulated DEGs. There were three DEGs that were downregulated upon the overexpression of every *edc* gene in Ea1189Δ8. Note that these results also include each of the *edc* genes themselves in each comparison if filtered through the statistical cutoff requirement. The venn diagram tool software (accessible at bioinformatics.psb.ugent.be/webtools/Venn/) was used to generate the venn diagram using RNAseq data.

A collective analysis of the transcriptomic patterns for Ea1189Δ8 vs. Ea1189Δ8 overexpressing each of the five *edc* genes indicated that a total of 121 DEGs (DESeq2 FDR cutoff 0.05 and a fold change of two (log2)) were present collectively among the five datasets (Fig 5A, Sup. Datasheet A). Plotting the data to check for any overlap of genes for the five comparisons showed that a majority of the genes among the overall DEGs were primarily regulated by one of the *edc* genes. An exception to this was Ea1189Δ8/*edcA* OE wherein the three (negatively regulated) genes that were filtered through the statistical analysis were the three common DEGs across all five datasets. These genes included *EAM_1085* (encodes for a leucine rich repeat domain containing protein), *EAM_3468* (encodes for a glycoside hydrolase family protein) and *EAM_2517* (unknown product) (Table 3). Relative to Ea1189Δ8, Ea1189Δ8/*edcB* OE had several metabolic and regulatory genes among the positive and negative DEGs including *yqaB*, *hrpA*, *hchA*, *phoH*, *hutG* and *rmf* (Table 4). Ea1189Δ8/*edcB OE* had the highest number of DEGs of all included comparisons with the other *edc*s in this study. Ea1189Δ8/*edcC OE* uniquely had *hslU*, *hslV* and EAM_RS16210 (encodes a zinc/ cadmium/ mercury/ lead-transporting ATPase), three ATPase related genes among the positive DEGs. Also, *dnaK*, *ibpA*, *casB* and *cas7e* were some regulatory targets related to housekeeping or type I CRISPR system (Table 5). Ea1189Δ8/*edcD* OE compared to Ea1189Δ8 only had significantly negative DEGs (barring the overexpression of *edcD* itself) including *casB*, EAM_RS14585 (encodes a aminotransferase phosphate dependent enzyme) and EAM_RS14565 (encodes a type I polyketide synthase) (Table 6). Ea1189Δ8/*edcE* OE relative to Ea1189Δ8 in addition to metabolic and regulatory targets had several genes related to the type I CRISPR system among the negative DEGs including *casB*, *cas7e*, *cas1e*, *cas5e*, *cas6e* and *casA* (Table 7). A complete list of all DEGs is provided in supplemental datasheet 1 and heighted DEGs for each individual comparative analysis is provide in Tables 3–7 The RNAseq study was validated through q-RT-PCR for a subset of genes (S1B Fig).

**Table 3:** A list of the statistically significant differentially regulated genes (Log2 fold change of two) in Ea1189Δ8+*edcA* OE relative to Ea1189Δ8.

Locus Tag	Gene ID	Product	Differential Expression Log2 Ratio	Differential Expression p-value
EAM_RS12275	hypothetical protein CDS	hypothetical protein	-2.046415814	2.69E-08
EAM_RS05285	leucine-rich repeat domain-containing protein CDS	leucine-rich repeat domain-containing protein	-2.22388315	1.14E-05
EAM_RS17045	glycoside hydrolase family 68 protein CDS	glycoside hydrolase family 68 protein	-2.76359249	7.20E-14

**Table 4:** A list of the 15 most positively and negatively regulated genes in Ea1189Δ8+*edcB* OE relative to Ea1189Δ8.

Locus Tag	Gene ID	Product	Differential Expression Log2 Ratio	Differential Expression p-value
EAM_RS02755	GGDEF domain-containing protein CDS	GGDEF domain-containing protein	10.52231065	6.1851E-189
EAM_RS15020	DEAD/DEAH family ATP-dependent RNA helicase CDS	DEAD/DEAH family ATP-dependent RNA helicase	3.837091548	1.16277E-13
EAM_RS16040	*glpD* CDS	glycerol-3-phosphate dehydrogenase	3.769007801	2.3349E-166
EAM_RS19305	*yrbN* CDS	protein YrbN	3.647272094	5.19945E-39
EAM_RS12870	HlyC/CorC family transporter CDS	HlyC/CorC family transporter	3.200198525	5.21317E-57
EAM_RS02090	pantoate—beta-alanine ligase CDS	pantoate—beta-alanine ligase	3.161465532	2.6906E-14
EAM_RS12890	*yqaB* CDS	fructose-1-phosphate/6-phosphogluconate phosphatase	2.939408173	6.28088E-17
EAM_RS19660	hypothetical protein CDS	hypothetical protein	2.875871177	2.25958E-29
EAM_RS15625	*rplF* CDS	50S ribosomal protein L6	2.766997946	2.05003E-08
EAM_RS12865	inner membrane protein YpjD CDS	inner membrane protein YpjD	2.740181732	1.89396E-14
EAM_RS18730	*yidD* CDS	membrane protein insertion efficiency factor YidD	2.706686489	2.12804E-30
EAM_RS19655	hypothetical protein CDS	hypothetical protein	2.678070834	1.05966E-15
EAM_RS15575	*rplQ* CDS	50S ribosomal protein L17	2.663892023	7.43101E-06
EAM_RS19535	hypothetical protein CDS	hypothetical protein	2.648755631	9.56953E-08
EAM_RS12880	glutamate—cysteine ligase CDS	glutamate—cysteine ligase	2.61623674	9.55405E-11
EAM_RS10630	hypothetical protein CDS	hypothetical protein	-3.779142872	8.15852E-22
EAM_RS14150	Hrp pili protein HrpA CDS	Hrp pili protein HrpA	-3.465753769	1.42198E-09
EAM_RS09925	*phoH* CDS	phosphate starvation-inducible protein PhoH	-3.304435872	3.67098E-10
EAM_RS04570	hypothetical protein CDS	hypothetical protein	-3.22780552	9.51376E-11
EAM_RS01050	dienelactone hydrolase family protein CDS	dienelactone hydrolase family protein	-3.105210459	3.22279E-25
EAM_RS05285	leucine-rich repeat domain-containing protein CDS	leucine-rich repeat domain-containing protein	-3.014341576	2.76182E-19
EAM_RS06155	*hutG* CDS	N-formylglutamate deformylase	-3.010799292	3.27597E-16
EAM_RS18215	*rmf* CDS	ribosome modulation factor	-2.962193193	1.30948E-08
EAM_RS18330	hypothetical protein CDS	hypothetical protein	-2.89078444	5.49473E-28
EAM_RS01820	*hchA* CDS	protein deglycase HchA	-2.858429374	8.44902E-16
EAM_RS02925	hypothetical protein CDS	hypothetical protein	-2.796397476	2.19042E-05
EAM_RS07845	hypothetical protein CDS	hypothetical protein	-2.788775685	2.46288E-13
EAM_RS09335	*yeaG* CDS	protein kinase YeaG	-2.784222226	6.44011E-12
EAM_RS17045	glycoside hydrolase family 68 protein CDS	glycoside hydrolase family 68 protein	-2.770732143	7.28882E-18
EAM_RS12275	hypothetical protein CDS	hypothetical protein	-2.729613743	1.53082E-27

**Table 5:** A list of the statistically significant differentially regulated genes (Log2 fold change of two) in Ea1189Δ8+*edcC* OE relative to Ea1189Δ8.

Locus Tag	Gene ID	Product	Differential Expression Log2 Ratio	Differential Expression p-value
EAM_RS07310	sensor domain-containing diguanylate cyclase CDS	sensor domain-containing diguanylate cyclase	7.11876968	1.87E-42
EAM_RS13595	*lysA* CDS	diaminopimelate decarboxylase	3.145640602	4.18E-15
EAM_RS16210	zinc/cadmium/mercury/lead-transporting ATPase CDS	zinc/cadmium/mercury/lead-transporting ATPase	3.127126824	5.96E-31
EAM_RS00635	*hslV* CDS	ATP-dependent protease subunit HslV	2.863823003	4.45E-16
EAM_RS03185	*dnaK* CDS	molecular chaperone DnaK	2.461525153	8.11E-10
EAM_RS16940	*ibpA* CDS	heat shock chaperone IbpA	2.403589754	8.31E-07
EAM_RS02090	pantoate—beta-alanine ligase CDS	pantoate—beta-alanine ligase	2.358062352	6.00E-09
EAM_RS15020	DEAD/DEAH family ATP-dependent RNA helicase CDS	DEAD/DEAH family ATP-dependent RNA helicase	2.3490175	6.18E-07
EAM_RS02145	co-chaperone GroES CDS	co-chaperone GroES	2.265795717	2.62E-09
EAM_RS00630	*hslU* CDS	HslU—HslV peptidase ATPase subunit	2.198704024	4.16E-31
EAM_RS15625	*rplF* CDS	50S ribosomal protein L6	2.162131775	2.28E-06
EAM_RS02100	cupin domain-containing protein CDS	cupin domain-containing protein	2.112275705	4.16E-16
EAM_RS05285	leucine-rich repeat domain-containing protein CDS	leucine-rich repeat domain-containing protein	-3.887406921	1.50E-37
EAM_RS14565	type I polyketide synthase CDS	type I polyketide synthase	-2.594131364	4.41E-13
EAM_RS17045	glycoside hydrolase family 68 protein CDS	glycoside hydrolase family 68 protein	-2.5793142	1.54E-16
EAM_RS12275	hypothetical protein CDS	hypothetical protein	-2.400248876	2.95E-17
EAM_RS03730	*casB* CDS	type I-E CRISPR-associated protein Cse2/CasB	-2.271501335	2.32E-29
EAM_RS14585	aminotransferase class III-fold pyridoxal phosphate-dependent enzyme CDS	aminotransferase class III-fold pyridoxal phosphate-dependent enzyme	-2.200310517	4.17E-09
EAM_RS14570	non-ribosomal peptide synthetase CDS	non-ribosomal peptide synthetase	-2.121916042	2.18E-07
EAM_RS18215	*rmf* CDS	ribosome modulation factor	-2.054007376	2.53E-05
EAM_RS03735	*cas7e* CDS	type I-E CRISPR-associated protein Cas7/Cse4/CasC	-2.035412707	1.38E-12

**Table 6:** A list of the statistically significant differentially regulated genes (Log2 fold change of two) in Ea1189Δ8+e*dcD* OE relative to Ea1189Δ8.

Locus Tag	Gene ID	Product	Differential Expression Log2 Ratio	Differential Expression p-value
EAM_RS10555	diguanylate cyclase CDS	diguanylate cyclase	5.778041665	2.25E-35
EAM_RS05285	leucine-rich repeat domain-containing protein CDS	leucine-rich repeat domain-containing protein	-3.266334894	4.90259E-27
EAM_RS17045	glycoside hydrolase family 68 protein CDS	glycoside hydrolase family 68 protein	-2.653853287	4.8117E-14
EAM_RS14565	type I polyketide synthase CDS	type I polyketide synthase	-2.363717088	4.30385E-10
EAM_RS03730	*casB* CDS	type I-E CRISPR-associated protein Cse2/CasB	-2.222582243	3.32759E-14
EAM_RS14585	aminotransferase class III-fold pyridoxal phosphate-dependent enzyme CDS	aminotransferase class III-fold pyridoxal phosphate-dependent enzyme	-2.146738285	1.27856E-09
EAM_RS12275	hypothetical protein CDS	hypothetical protein	-2.093779402	1.12176E-10
EAM_RS14570	non-ribosomal peptide synthetase CDS	non-ribosomal peptide synthetase	-2.037259516	1.01262E-07

**Table 7:** A list of the statistically significant differentially regulated genes (Log2 fold change of two) in Ea1189Δ8+*edcE* OE relative to Ea1189Δ8.

Locus Tag	Gene ID	Product	Differential Expression Log2 Ratio	Differential Expression p-value
EAM_RS11860	sensor domain-containing diguanylate cyclase CDS	sensor domain-containing diguanylate cyclase	12.50422101	0
EAM_RS00635	*hslV* CDS	ATP-dependent protease subunit HslV	2.83447676	4.41E-10
EAM_RS02145	co-chaperone GroES CDS	co-chaperone GroES	2.689831641	1.23E-11
EAM_RS16940	*ibpA* CDS	heat shock chaperone IbpA	2.640983916	3.20E-07
EAM_RS03185	*dnaK* CDS	molecular chaperone DnaK	2.514067921	1.52E-09
EAM_RS06745	*hspQ* CDS	heat shock protein HspQ	2.376852632	3.70E-07
EAM_RS12775	*clpB* CDS	ATP-dependent chaperone ClpB	2.303745988	3.52E-07
EAM_RS14360	PTS sugar transporter subunit IIB CDS	PTS sugar transporter subunit IIB	2.254006979	5.91E-14
EAM_RS00630	*hslU* CDS	HslU—HslV peptidase ATPase subunit	2.186164935	1.37E-27
EAM_RS16210	zinc/cadmium/mercury/lead-transporting ATPase CDS	zinc/cadmium/mercury/lead-transporting ATPase	2.142197384	6.38E-17
EAM_RS03205	*rpsT* CDS	30S ribosomal protein S20	2.067365283	6.13E-05
EAM_RS15625	*rplF* CDS	50S ribosomal protein L6	2.009381497	3.20E-05
EAM_RS05285	leucine-rich repeat domain-containing protein CDS	leucine-rich repeat domain-containing protein	-4.528529394	1.91193E-44
EAM_RS17045	glycoside hydrolase family 68 protein CDS	glycoside hydrolase family 68 protein	-3.584785314	3.81385E-22
EAM_RS14565	type I polyketide synthase CDS	type I polyketide synthase	-3.20797224	8.07891E-25
EAM_RS14570	non-ribosomal peptide synthetase CDS	non-ribosomal peptide synthetase	-2.836841807	4.9235E-17
EAM_RS14585	aminotransferase class III-fold pyridoxal phosphate-dependent enzyme CDS	aminotransferase class III-fold pyridoxal phosphate-dependent enzyme	-2.62716761	6.16167E-19
EAM_RS14575	KR domain-containing protein CDS	KR domain-containing protein	-2.591188195	1.74194E-11
EAM_RS03730	*casB* CDS	type I-E CRISPR-associated protein Cse2/CasB	-2.403157946	1.80146E-22
EAM_RS12275	hypothetical protein CDS	hypothetical protein	-2.300109076	2.83284E-13
EAM_RS08495	isocyanide synthase family protein CDS	isocyanide synthase family protein	-2.273812019	1.45024E-12
EAM_RS03735	*cas7e* CDS	type I-E CRISPR-associated protein Cas7/Cse4/CasC	-2.173201222	2.00038E-13
EAM_RS06125	urocanate hydratase CDS	urocanate hydratase	-2.16671334	1.3265E-10
EAM_RS14590	hypothetical protein CDS	hypothetical protein	-2.155367805	8.86444E-05
EAM_RS14580	polyketide synthase CDS	polyketide synthase	-2.142735466	1.48983E-11
EAM_RS03750	*cas1e* CDS	type I-E CRISPR-associated endonuclease Cas1e	-2.077795929	8.24188E-11
EAM_RS03740	*cas5e* CDS	type I-E CRISPR-associated protein Cas5/CasD	-2.058059128	2.1109E-11
EAM_RS03745	*cas6e* CDS	type I-E CRISPR-associated protein Cas6/Cse3/CasE	-2.054121085	5.56701E-13
EAM_RS03725	*casA* CDS	type I-E CRISPR-associated protein Cse1/CasA	-2.049727209	7.12272E-20
EAM_RS06130	*hutH* CDS	histidine ammonia-lyase	-2.035079931	6.4966E-08
EAM_RS14560	type I polyketide synthase CDS	type I polyketide synthase	-2.033125808	2.43973E-13

## Discussion

Cyclic-di-GMP dependent regulation has been studied in many bacterial systems, and the results have highlighted various signaling roles for the second messenger ranging from phase transition to bacteriophage interactions [1,2,26]. A common impediment for studying the systematic impact of the genetic components of c-di-GMP metabolism has been the sheer multiplicity of the included elements [1,32,33]. In this regard, *E*. *amylovora* serves as a particularly useful pathogenic model because this organism encodes a fairly condensed set of Dgc and Pde enzymes in its c-di-GMP repertoire, and c-di-GMP regulates all of the most critical virulence factors that facilitate systemic movement through the host and disease progression [3,4].

Attachment and host xylem colonization are entirely dependent on c-di-GMP in *E*. *amylovora*. The recovery in surface attachment upon the restoration of any of the *edc* genes in Ea1189Δ12 signified that the surface interaction was c-di-GMP dependent in a quantitative sense. Further, the type IV pilus was an important determinant of surface attachment and required the flagellar filament as a mediator. Independent of c-di-GMP presence, *hofC* function was a limiting factor in terms of the ability of cells to attach to surface. However, the dependence on surface sensing through the flagellar filament was more prominent under lower intracellular levels of c-di-GMP. A similar co-dependence on both the flagellum and the pilus for surface attachment has been demonstrated in *P*. *aeruginosa*, wherein, upon first surface contact by the flagellar filament, the rotational changes sensed by the motor proteins MotAB result in a rapid increase in c-di-GMP levels which can bind to the effector FimW and regulate type IV pilus-based surface interaction [34]. We highlight that the regulation of attachment can occur through c-di-GMP generated by any of the five Edc sources, making this factor a global target of c-di-GMP generated within *E*. *amylovora* cells. Interestingly however, flagellar motility *in vitro* was not significantly altered in Ea1189Δ12 relative to Ea1189. In similar genetic reductionist studies aimed at understanding the impact of c-di-GMP on multiple phenotypes conducted in Salmonella and *Caulobacter crescentus*, a c-di-GMP null condition has been associated with altered motility *in vitro* [5,10]. Since flagellar motility is not a limiting factor in the shoot blight infection model [35], further studies will be necessary to link the impact of c-di-GMP on motility during other stages in the disease cycle.

Contact with a surface during biofilm initiation and the underlying c-di-GMP based regulation had global transcriptomic implications in *E*. *amylovora* with over 500 DEGs between surface exposed Ea1189 and Ea1189Δ12. A limitation of this study is the exclusive use of *in vitro* surface exposure treatments as opposed to studying the interactive dynamics of the pathogen with the host in planta. Transcriptomic studies conducted in *P*. *syringae* and *Ralstonia solanacearum* have revealed the complexity of the host dependent response occurring in bacterial phytopathogens [36,37]. A major physical limitation in our study was the inability of Ea1189Δ12 to colonize the xylem vessels which resulted in us having to rely on *in vitro* surface exposure using flow cells to be able to collect consistent and high quality RNA samples from our strains, not limited by the abundance of bacterial titer in planta. In this transcriptomic study, genes negatively expressed in Ea1189Δ12 vs. Ea1189 are indicative of targets that are positively regulated by c-di-GMP. Some of the critical regulatory genes in this category were *hmsP* (biofilm formation regulator) and EAM_RS05285 (leucine rich repeat (LRR) protein encoding gene). HmsP has been reported to be a negative regulator of biofilm formation in *Yersinia pestis* [38]. While LRR- family proteins are heavily involved in regulating plant bacterial resistance interactions, their link with c-di-GMP has been documented in animal immunogenic interactions involving the STING pathway, specifically by NLRC3 which can get activated by c-di-GMP and feed into the STING trafficking [39,40]. Conversely, genes negatively regulated by c-di-GMP during surface interactions included several type III (T3SS) and type VI secretion system (T6SS) related genes. Our previous work in *E*. *amylovora* has indicated that elevated intracellular levels of c-di-GMP can negatively regulate the T3SS transcriptionally [4]. While the impact of the T6SS linked to c-di-GMP has not been explored in *E*. *amylovora*, in *P*. *aeruginosa* the T6SS protein TfoY can be partially triggered by reduced intracellular levels of c-di-GMP and can lead to altered levels of bacterial killing mediated by the T6SS [41]. Also in *P*. *aeruginosa*, the RetS/GacA sensor protein can respond to changing c-di-GMP levels and mediate the switching between the T3SS and T6SS [42]. Overall, these collective targets are indicative of an evolutionary bottleneck making c-di-GMP a limiting factor for host colonization. A similar transcriptomic study in *Pseudomonas syringae* heterologously overexpressing a Dgc or a Pde revealed that the altered levels of c-di-GMP can impact several genes related to flagellar structure and function, as well as those involved in chemotaxis, metabolism and two component system transduction [43]. The relatively under-researched aspect of our findings is perhaps the multiple positively and negatively regulated metabolic targets dependent on c-di-GMP. Further studies will be required to document the pathway specific impacts of c-di-GMP during biofilm initiation. Our study highlights that surface sensing and biofilm initiation are heavily dependent on c-di-GMP in *E*. *amylovora* and require the successful transcriptional regulation of a large network of genes.

In *E*. *amylovora*, biofilm formation was a globally impacted by multi-sourced c-di-GMP. In *Salmonella*, Solano et al. found that four of the twelve Dgcs were involved in cellulose production, and other aspects related to virulence and biofilm formation [5]. The regulatory model under which multiple Dgcs regulate one phenotype is widely present in other pathosystems including *Escherichia coli*, *P*. *aeruginosa* and *V*. *cholerae* [7,9,33,44–47]. T3SS expression, quantified via *hrpL* transcription, was significantly elevated in Ea1189Δ12, which corroborates the existing model of c-di-GMP driven negative regulation of T3SS in *E*. *amylovora* [3,4]. Disease progression in apple shoots is dependent on both the T3SS in the apoplast and biofilm formation in the xylem vessels [11,35]. Thus, despite the high levels of *hrpL* expression in Ea1189Δ12, this strain was unable to colonize the xylem or cause shoot blight. This also holds true for Ea1189Δ12 complemented with individual *edc* genes wherein higher than WT levels of *hrpL* expression were recorded in all five strains but this did not result in significant changes in virulence in planta (barring *edcE*). While our *in vitro* biofilm assessment indicated that all Edcs could regulate biofilm formation in flow cells, EdcE was the only diguanylate cyclase that enabled Ea1189Δ12 to regain the ability to colonize the xylem as inferred through the progression of shoot blight for these strains. This indicates that phenotypic switching and attachment within the xylem could have evolved to be mainly dependent on EdcE over the other Edcs in *E*. *amylovora*. Contrastingly in Ea1189Δ8, it is unclear if the most significant contributor to the observed reduction in virulence is the reduced *hrpL* expression, the impairment in surface attachment or both. The observation of differential *hrpL* expression in Ea1189Δ8 compared to Ea1189Δ12 highlights the potential importance of the four degenerate GGDEF/EAL proteins (EAM_3378, CsrD, EAM_2449, EAM_1579) in regulating the T3SS. Evidence suggests that proteins with degenerate GGDEF/EAL domains can serve as c-di-GMP binding receptors and regulate diverse function [48,49]. In *E*. *amylovora*, CsrD can regulate *amsG* transcription by binding to c-di-GMP and modifying the degradation efficacy of RNase E towards the small RNA CsrB [15]. However, the role of these degenerate GGDEF/EAL domain containing proteins is not fully understood in the context of *hrpL* transcription. Thus, the use of Ea1189Δ8 provided us the ability to study the impact of c-di-GMP metabolism without the interruption of any signaling activity from other potential c-di-GMP effectors.

In addition to the targeted evaluation of the effects of c-di-GMP on virulence factors, we also took an untargeted approach through RNAseq to understand the global transcriptomic map dependent on each of the Edcs. Most of the DEGs affected by each individual Edc were unique and only a small subset were co-regulated by two or more Edcs. A general mix of metabolic, regulatory and structural genes were among the DEGs. The largest number of DEGs were impacted by EdcB, however further investigation is required to implicate this effect on increased c-di-GMP generation or the presence of multiple c-di-GMP targets channeled through this enzyme. The phylogenies of all the Edcs in *E*. *amylovora* don’t show signs of very recent acquisition, which could suggest that the spatial effect of each of the Edcs is due to some level of evolution-driven host adaptation [50,51].

Among all the *edc* overexpression based transcriptomic datasets, a shared factor was the reduced expression of EAM_RS05285 (encodes for a leucine rich repeat domain containing protein) upon the overexpression of each of the *edc* genes, implying that this gene is negatively regulated by c-di-GMP. However, in the other transcriptomic experiment involving surface exposed Ea1189 and Ea1189Δ12 cells also included in this study, the same gene was found to be positively regulated by c-di-GMP. From this we are able to infer that the regulation of EAM_RS05285 is dependent on the state of the cell and if the cell contacts a surface and on c-di-GMP production within the cell from any *edc* source.

Apart from *edcA*, the overexpression of each of the four *edc* genes (*edcB-E*) revealed unique regulatory targets. Interestingly the number of differentially regulated genes and the proportion of positively and negatively regulated genes varied in each case as well. For *edcB*, *edcC* and *edcD* this comprised of regulatory and metabolic genes. With the overexpression of *edcE* in Ea1189Δ8, several genes involved in the type I CRISPR system were negatively regulated. While *casB* and *cas7e* were also differentially regulated when *edcC* and *edcD* were overexpressed in the same background, the effect became more pronounced with the overexpression of *edcE*, which had an impact on six CRISPR related genes. CRISPR elements have been used in genotypically categorizing and tracking pathogenic evolution in *E*. *amylovora* [52–54]. The type I CRISPR system in *E*. *amylovora* was recently reported to be involved in resistance against invasive plasmids and not necessarily against phages [55]. No link has been identified to c-di-GMP regulation in this regard. In terms of regulatory targets, *edcB* had mostly metabolic targets represented among the top DEGs. *hrpA* was also downregulated significantly by the overexpression of *edcB*. While c-di-GMP is known to reduce T3SS expression in *E*. *amylovora* from previous studies [3,4], specificities regarding the generative source of the c-di-GMP have not been identified. Further, our phenotypic data measuring virulence and *hrpL* expression highlights *edcC* and *edcE* as being the significant contributors to T3SS when complemented in Ea1189Δ12. While these two sets of experiments differ based on the overexpression or native promoter driven expression of the *edc* genes, the overall results highlight that T3SS regulation in *E*. *amylovora* is dependent on c-di-GMP on a spatiotemporal basis. The overexpression of *edcC* and *edcE* resulted in the increased expression of heat shock related proteins including *ibpA*, *dnaK* and *hspQ*. Lon and CsrA have been linked to heat shock response regulation in *E*. *amylovora* and the heat shock response of the pathogen is a target for AvrRpt2 mediated resistance to *E*. *amylovora* in apple [56–59]. While the link between heat shock response and c-di-GMP has not been studied in *E*. *amylovora*, in *V*. *cholerae*, Lon was identified as a c-di-GMP receptor that could regulate the stability of TfoY leading to downstream effects on the heat shock response of the pathogen [60]. Thus, our study highlights the complexity of c-di-GMP signaling and the evolutionary importance of utilizing multiple c-di-GMP generative Dgcs in a given pathogenic system. While the distribution of c-di-GMP can be variable depending on the state of the cell and the location within the cell, as previously documented in *Caulobacter crescentus* [25], further evidence will be needed to document the temporal origin of c-di-GMP from a specific *edc/dgc* source and its direct impact on specific genetic targets.

**Fig 6:**
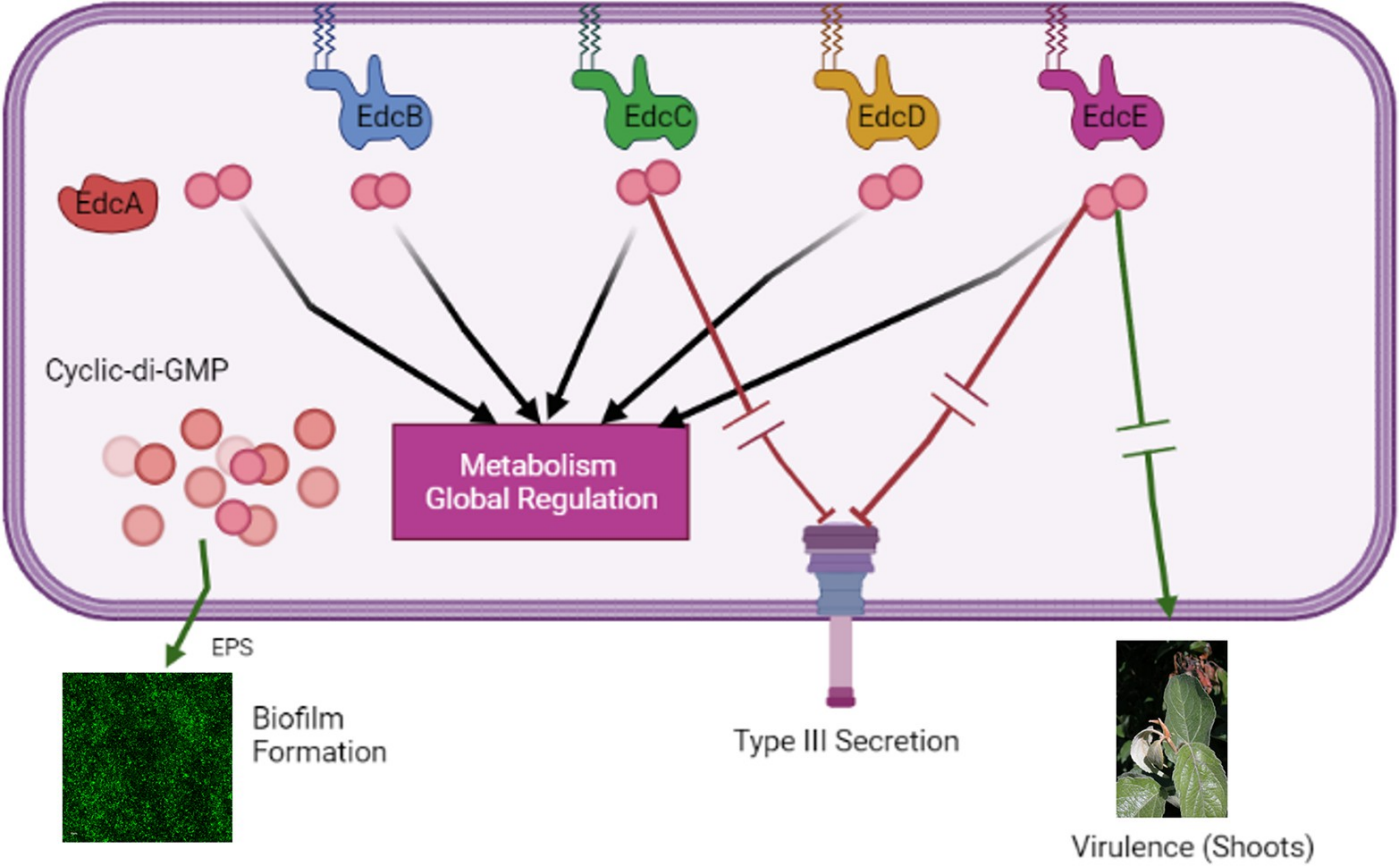
C-di-GMP regulatory model in surface exposed *E*. *amylovora* cells. Our study indicates that there is a dimorphism in the regulatory targets of the c-di-GMP generated by each of the five Edcs. While each Edc uniquely regulates the transcription of several genes, and virulence factors *in vitro* and in planta, attachment/biofilm formation (dependent on EPS production) is regulated by all the Edcs, thus, leading us to hypothesize about the potential presence of a localized and a diffused pool of c-di-GMP that can achieve these varied regulatory targets. Red inhibitor lines and green arrows indicate negative and positive regulation respectively, with arrow breaks indicating intermediate regulatory steps.

In *E*. *amylovora*, we hypothesize that the c-di-GMP generated by each of the Edcs can have both localized effects that are unique to each of the Edcs and also that diffused pool/s of c-di-GMP that can be generated through contributions of multiple or all Edcs. A diffused pool of c-di-GMP could be used to regulate some of the shared factors that are impacted by all the Edcs (Fig 6). While our study has focused on c-di-GMP dependent regulation, we are left with some critical unanswered questions about the degradation of c-di-GMP once it has been generated by an Edc enzyme. All three active Pdes in this system are anchored in the cytoplasmic membrane, which could affect their ability to degrade c-di-GMP from varied Edc sources as well as any diffused intracellular pool of c-di-GMP [26]. Further, this raises a consideration that during heterologous expression of a non-native Dgc, an approach which is often used to alter c-di-GMP levels and check the phenotypic impact, the results could be skewed by the presence/absence of localized effects. For such future investigations, we will be able to use Ea1189Δ12 as an ideal background strain to study the localization and generation/hydrolysis dynamics of c-di-GMP within the cell.

## Materials and methods

### Bacterial strains, plasmids and growth conditions

All bacterial strains, and plasmids used in this study along with their relevant characteristics are described in Table 8. Unless specified otherwise, *E*. *amylovora* strains were grown in Lysogeny broth (LB) amended with one or more of the appropriate antibiotics: ampicillin (Ap; 100 μg/ml), chloramphenicol (Cm; 10 μg/ml), gentamicin (Gm; 10 μg/ml) or kanamycin (Km; 100 μg/ml). Overexpression constructs (*hofC* OE and *fliC* OE) were induced using 1 mM isopropyl-b-D-thiogalactopyranoside (IPTG).

**Table 8:** Strains, plasmids and relevant information.

Strain/Plasmid	Relevant Characteristics	Source
*E*. *amylovora* strains		
Ea1189	Wild Type	[3]
Ea1189Δ8	Deletion of *edcA-E* and *pdeA-C*	This study
Ea1189Δ12	Deletion of *edcA-E*, *pdeA-C*, EAM_3378, EAM_3136, EAM_2449 and EAM_1579	[72]
Ea1189Δ12Δ*fliC*	Deletion of *fliC* in Ea1189Δ12	This study
Ea1189Δ12Δ*hofC*	Deletion of *hofC* in Ea1189Δ12	This study
Ea1189Δ*fliC*	Deletion of *fliC* in Ea1189	This study
Ea1189Δ*hofC*	Deletion of *hofC* in Ea1189	This study
Ea1189Δ12 + *edcA-E*	Chromosomal restoration of the indicated gene in Ea1189Δ12	This study
Plasmids		
pKD3	Cm^r^ cassette flanking FRT* sites; Cm^r^	[61]
pKD4	Km^r^ cassette flanking FRT sites; Km^r^	[61]
pKD46	L-Arabinose-inducible lambda red recombinase; Ap^r^	[61]
pTL18	IPTG-Inducible FLPase, Tet^R^	[73]
pBBR1-MCS5	Broad-host-range cloning vector**; R6K ori; Gm^r^	[74]
pMP2444	pBBR1MCS-5 expression *gfp* under *lac* promoter, Gm^r^	[75]
pEVS143	Broad-host-range, IPTG inducible (Ptac) cloning vector; inducible Cm^r^ and GFP Km^r^	[76]
*hofC* OE	*hofC* in pEVS143	This study
*fliC* OE	*fliC* in pEVS143	This study
*edcA* OE	*edcA* in pEVS143	[3]
*edcB* OE	*edcB* in pEVS143	[3]
*edcC* OE	*edcC* in pEVS143	[3]
*edcD* OE	*edcD* in pEVS143	[3]
*edcE* OE	*edcE* in pEVS143	[3]

*FRT: Flippase target recognition

**MCS: Multiple cloning site.

### Genetic manipulation and bioinformatics

Reference genome sequence for *E*. *amylovora* Ea1189 (Accession: FN434113) was obtained from NCBI. Artemis (Java) was used to browse the genome. The standard lambda Red recombinase protocol was used to construct chromosomal deletion mutants [61]. To complement the individual deleted genes, the full-length sequences of the genes were briefly amplified, and the purified gene fragments were transformed into Ea1189Δ12 harboring pKD46 (induced with arabinose) along with a retained Cm^R^/Km^R^ cassettes (originally amplified from pKD3/pKD4 source plasmids) in the target gene being restored. Transformed cells were recovered after an 18 h incubation and were screened for a loss of the resistance cassette, followed by Sanger sequencing to confirm the replacement of the originally deleted gene.

### Scanning electron microscopy and bacterial population quantification to monitor xylem colonization in apple shoots

Apple shoots were inoculated as previously described [3]. Strains were grown overnight at 28°C and normalized to an OD_600_ of 0.2. Scissors dipped in inoculum were used to make an incision between two peripheral veins on young apple leaves (*Malus x domestica* cv. Gala on M9 rootstock). At 3 dpi, inoculated leaves along with the attached petiole were harvested. Cross sections of the petioles and apoplast tissue were fixed using 2.5% paraformaldehyde-2.5% glutaraldehyde, followed by ethanol dehydration at increasing concentrations as previously described [13]. Samples were imaged using the JEOL 6610LV (Japan Electron Optics Laboratory Ltd., Tokyo, Japan).

In order to quantify the level of bacterial proliferation and movement in apple shoots, the inoculated leaves and petioles were collected from infected shoot tips at 0, 1, 2 and 3 dpi, weighed and crushed in 0.5X phosphate buffered saline (PBS) solution. Serial dilutions were used to determine the population counts for WT Ea1189 and Ea1189Δ12, which were normalized by tissue weight. This study involved three biological replicates. JMP statistical software was used for data analysis.

### Growth curve analysis

Strains were grown overnight in LB amended with antibiotics as appropriate. Following this, the strains were sub-cultured in individual wells on a 96 well plate and were adjusted to a starting OD_600_ of ~0.1 in LB media. This setup was then incubated for 19 hrs in a TECAN Spark spectrophotometer (Tecan, Männedorf, Switzerland) at 28°C, with OD_600_ measurements taken every hour. This study involved three biological replicates. JMP statistical software was used for data analysis.

### Confocal microscopy to monitor attachment and biofilm formation in flow cells

To monitor initial surface interaction and attachment, *E*. *amylovora* strains expressing pMP2444::*gfp* [13] were grown for 18 h at 28°C and normalized to an OD_600_ of 0.5. A total of 1 ml of inoculum for each strain was introduced into a flow cell chamber in a μ-Slide VI 0.5 glass bottom slide (Ibidi, Martinsried, Germany). Immediately, the base of the flow chamber was repeatedly imaged using a Olympus FlouView 1000 confocal laser scanning microscope (Olympus, MA, USA). Images were acquired for up to 1 h or until the frame was saturated with fluorescent cell signals. Following this, the flow cell chamber was flushed with 5 ml of 0.5X phosphate buffered saline (PBS). To evaluate biofilm formation, following the initial attachment incubation, the flow chamber was subjected to flow (0.5X LB) using a peristaltic pump (Ismatec REGLO Digital 4-CH pump) (Cole-Parmer IL, USA) for 5 h. Fluorescent Z-stacked images were acquired to measure overall attachment and biofilm levels in the flow cell chambers [13]. ImageJ software was used to invert the color on the images, and the RBG plugin was used to process these images and to graph the GFP signal intensity profile for the Z-stacked images [62].

### Quantifying intracellular levels of c-di-GMP

Intracellular levels of c-di-GMP were quantified as previously described [3]. Strains were grown in overnight in LB, sub-cultured, collected at mid-log phase and lysed (with 40% acetonitrile and 40% methanol) at -20°C for 1 h. Relative levels of c-di-GMP in the samples was established against a standard curve generated using synthesized c-di-GMP (Axxora Life Sciences Inc., CA, USA) using a Quattro Premier XE instrument (Waters Corp. MA, USA). Three biological replicates were included in the studies. JMP statistical software was used for data analysis.

### q-RT-PCR to measure gene expression

To measure *hrpL* expression, strains were grown overnight in LB at 28°C. Cell cultures were then washed and resuspended in HRP-MM medium and incubated for 6 h [4]. To validate RNA-Seq data, identical sample treatment and collection protocols were used, with the exception of measuring transcript levels only for representative gene targets. RNA extraction and concentration for all samples were conducted using the protocols described for RNA-Seq sample collection. To check the impact of c-di-GMP on attachment appendages gene expression, including *fliC*, *hofC*, *crl* and *fimA*, WT Ea1189 and Ea1189Δ12 were incubated in a flow cell chamber for 1 h, thus resembling the treatment experienced by the surface treated strains included in the RNAseq experiment. Following surface exposure, the cells were collected from the flow chamber and RNA was extracted using the protocol by Rivas et. al., similar to the protocol for RNAseq sample extraction [63]. cDNA was synthesized using the High Capacity RT kit (Applied Biosystems, CA, USA). SYBR green PCR master mix (Applied Biosystems, CA, USA) was used for quantitative PCR experiments. *recA* was used as an endogenous control. The delta C_T_ method was used to compare transcriptomic fold changes [64]. Three biological replicates were included in the studies. JMP statistical software was used for data analysis.

### Virulence assays

Relative virulence levels of strains were compared using apple shoots as previously described [3]. Apple shoots were inoculated using the same described protocol used to evaluate xylem colonization through SEM. Data was collected in the form of necrotic lesion length along the shoot 8 dpi. JMP statistical software was used for data analysis.

### Flagellar motility assay

Relative levels of flagellar motility were quantified *in vitro* using a previously described protocol [4]. Strains were grown overnight in LB at 28°C. Following this, the OD_600_ for the cultures was normalized to 0.5. Cultures were stab inoculated onto a motility agar plate and incubated for 48h. Motility was quantified in terms of the diameter of the colony movement using ImageJ software [62]. JMP statistical software was used for data analysis.

### RNA-Seq sample acquisition, sequencing, data analysis and overall data integration/modelling

WT *E*. *amylovora* Ea1189 and Ea1189Δ12 were grown overnight in LB medium, sub-cultured and harvested at the mid-log phase prior to RNA extraction. To collect cell samples from reflective of the stages of biofilm development, the protocol described in this study to monitor biofilm formation in flow cells remained largely the same (note that the strains were not fluorescently labelled). Inoculum injected into the flow chamber for the strains was collected 1 h after the cells were allowed to interact with the surfaces in the flow chamber before being collected for RNA extraction. For the other comparative RNAseq study presented in the study, Ea1189Δ8 and Ea1189Δ8 complemented with overexpression vectors for *edcA-E* individually were all grown overnight in LB medium, followed by a sub-culturing, IPTG (1mM) induction as appropriate and sample collection at mid-log phase at which stage they were processed for RNA extraction. Three biological replicates were included in the study.

For RNA extraction, as per a previously described protocol [63], cells were washed with 0.1% N-lauryl sarcosine sodium salt, followed by treatment with the lysis buffer (1% SDS in 10 mM EDTA and 50 mM sodium acetate, pH 5.1) and a 5 min incubation in boiling water. The extracted RNA was treated for residual DNA contamination using the TURBO DNA-free kit (Thermo Fisher Scientific, MA, USA), and concentrated using the RNA Clean and Concentrator-25 kit (Zymo Research, CA, USA). The ‘Unbound’ samples were treated after their collection from the flow chamber, for the other two sample types, the initial lysis and wash steps were conducted by injecting the buffers directly into the flow chamber and suctioning the fluid out.

RNA samples were analyzed for quality control on the Agilent 4200 TapeStation (Agilent Technologies, CA, USA). The QIAseq FastSelect 5S/16S/23S rRNA removal kit was used to treat samples prior to library prep with the TruSeq Stranded Total RNA Library Prep Kit (Illumina, CA, USA). Sequencing was conducted on the Illumina HiSeq 4000 at 50 bp single-end reads.

For data analysis, adaptor barcodes were filtered using Trimmomatic v 0.36 (single end criteria: ILLUMINACLIP:TruSeq3-SE:2:30:10 LEADING:3 TRAILING:3 SLIDINGWINDOW:4:15) [65]. Trimmed sequences were mapped to the *E*. *amylovora* ATCC-49946 genome using Bowtie v 2.4.1. HTSeq v 0.11.2 [66,67]. Differential expression analysis was conducted using DESeq2 v 3.12 with an FDR cutoff of 0.05 and a minimum accepted fold change of 2 (log2) [68]. Geneious software was used for volcano plot generation. Gene ontology enrichment analysis was conducted using BiNGO on the Cytoscape platform [69,70]. Biological GO enrichment was assessed with an FDR cutoff of 0.01.

For phylogenetic analysis of each of each of the Edcs, BLASTp program from NCBI was used with the parameters set to searching within the entire non-redundant protein database, with a cutoff threshold of 1e-05 using the BLOSUM62 matrix with conditional compositional score matrix adjustment (Existence 11 Extension 1). We then acquired the fast minimum evolution phylogenetic tree generated for the top 250 matches and compiled them for all the twelve genes in our study for collective analysis [71].

## Supporting information

S1 Data All data analyzed in this study. (XLSX)

S1 Datasheet All RNA-Seq data analyzed in this study. (XLSX)

S2 Datasheet Phylogenetic analysis of all Edcs in *E*. *amylovora*. (PDF)

S1 Fig Graph summarizing the RNAseq and q-RT-PCR based examination of fold changes in the expression of representative genes (from both RNAseq studies) A) atpG and fliG in Ea1189Δ12 relative to WT Ea1189 and B) EAM_2517 and EAM_1085 in Ea1189Δ8 over expressing *edcA-E* individually relative to Ea1189Δ8. Error bars represent standard error of the means.
(TIF)

S1 Video Surface attachment dynamics of WT Ea1189 conducted using confocal microscopy. (MP4)

S2 Video Surface attachment dynamics of Ea1189Δ12 conducted using confocal microscopy. (MP4)
