# Supplementary figures and images for: A complete twelve-gene deletion null mutant reveals that cyclic di-GMP is a global regulator of phase-transition and host colonization in Erwinia amylovora
Source: PLoS Pathog. 2022 Aug 1;18(8):e1010737. doi: 10.1371/journal.ppat.1010737 (PMC9371280; doi:10.1371/journal.ppat.1010737)

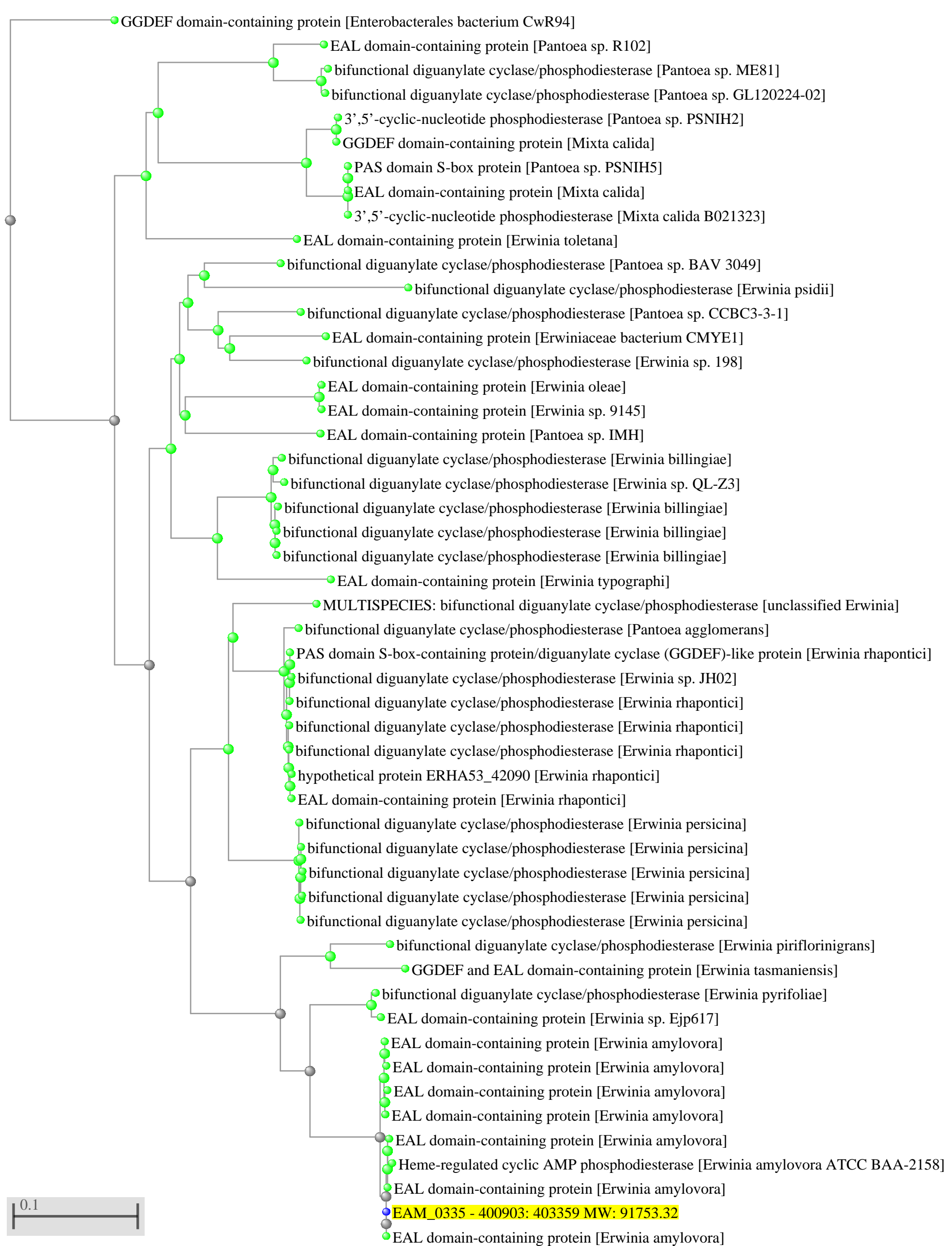

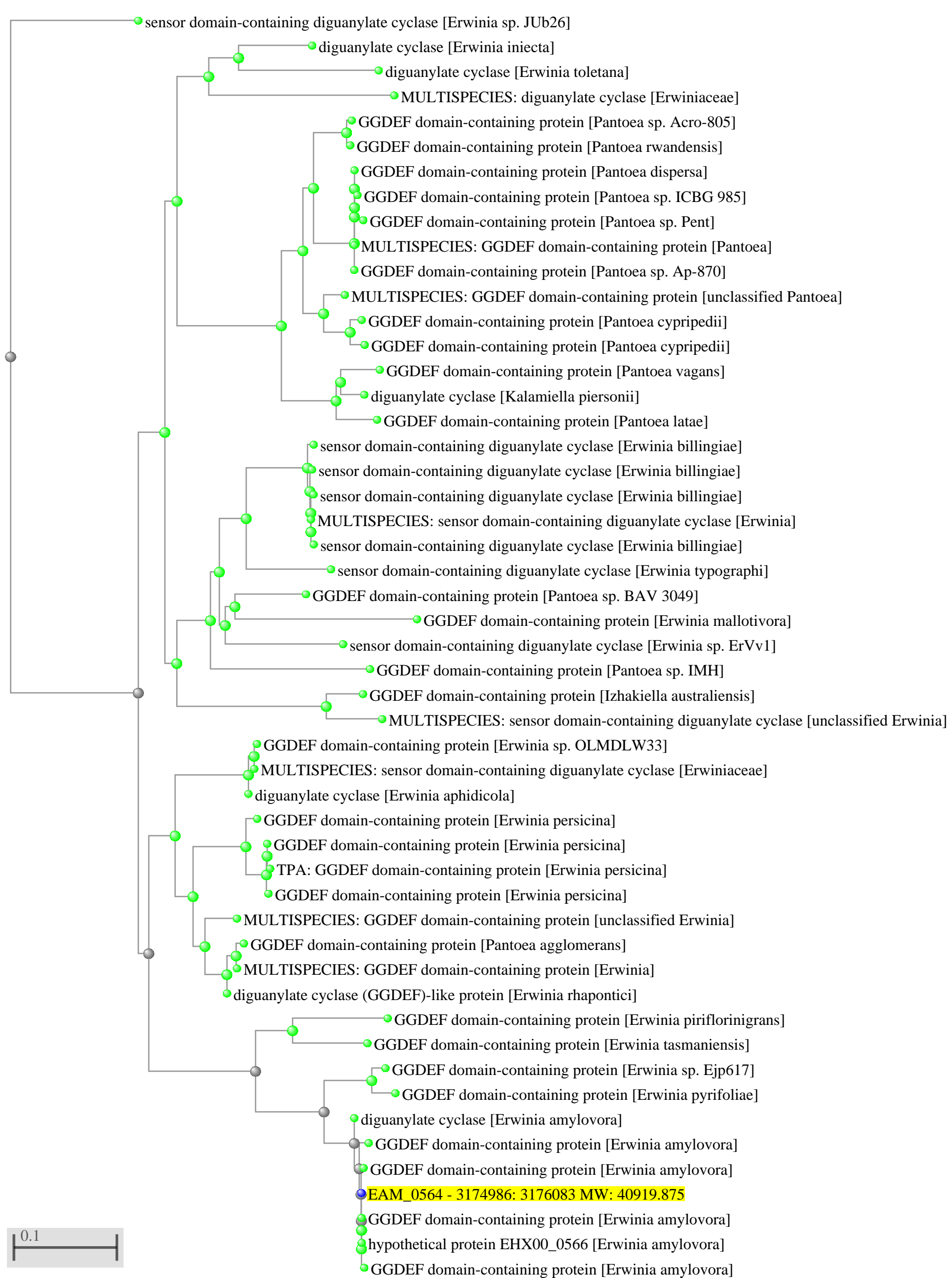

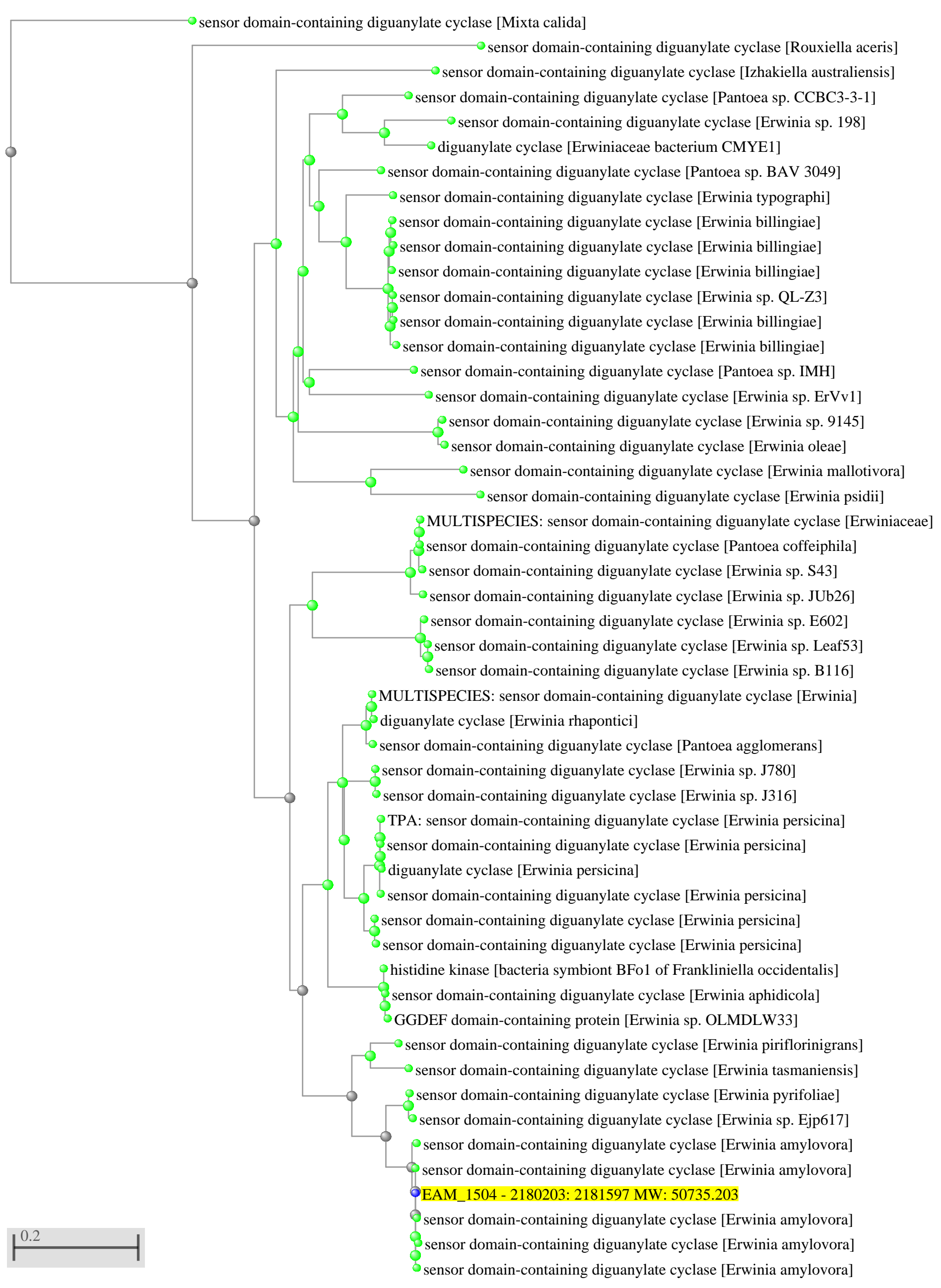

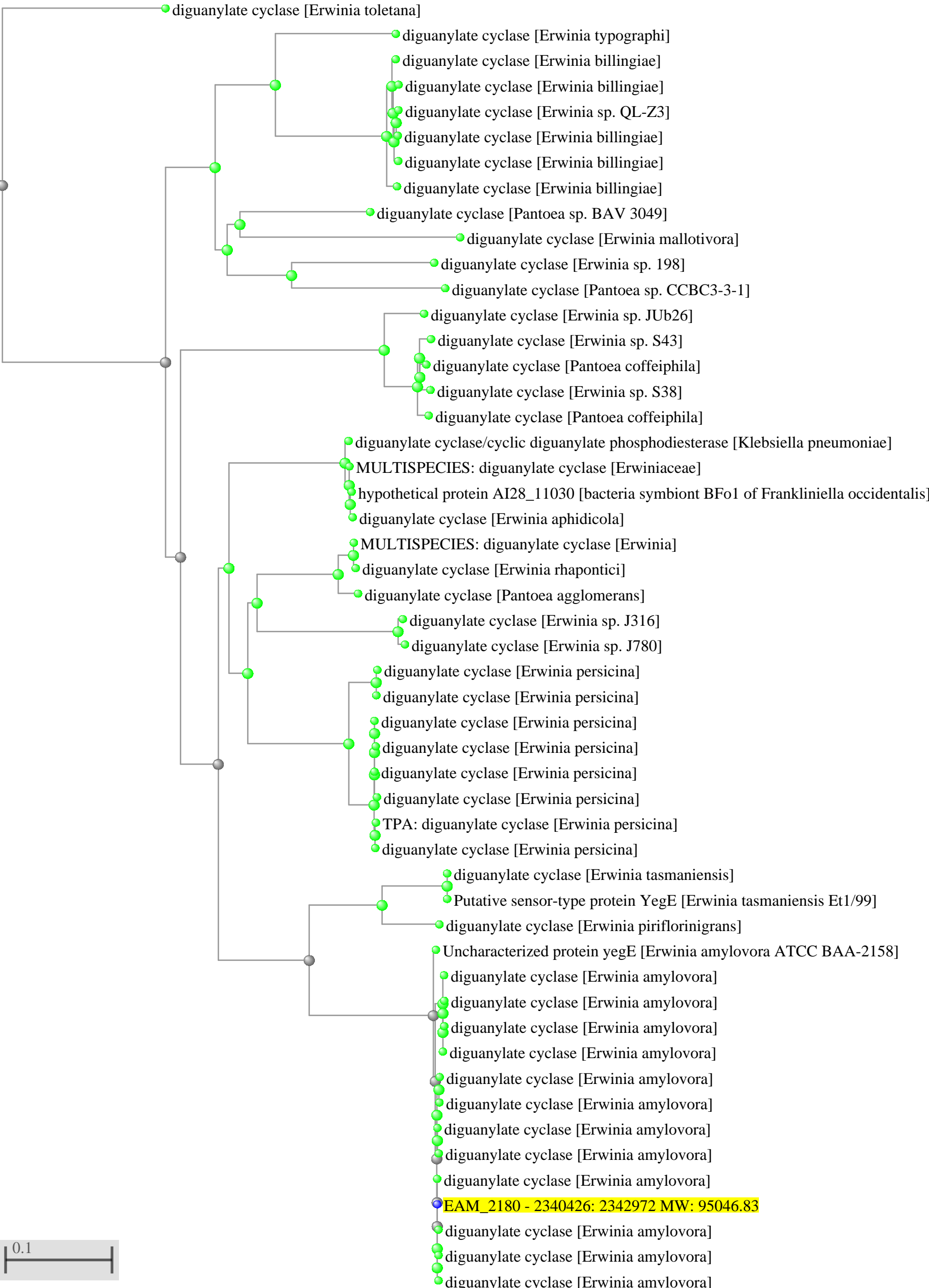

0.1

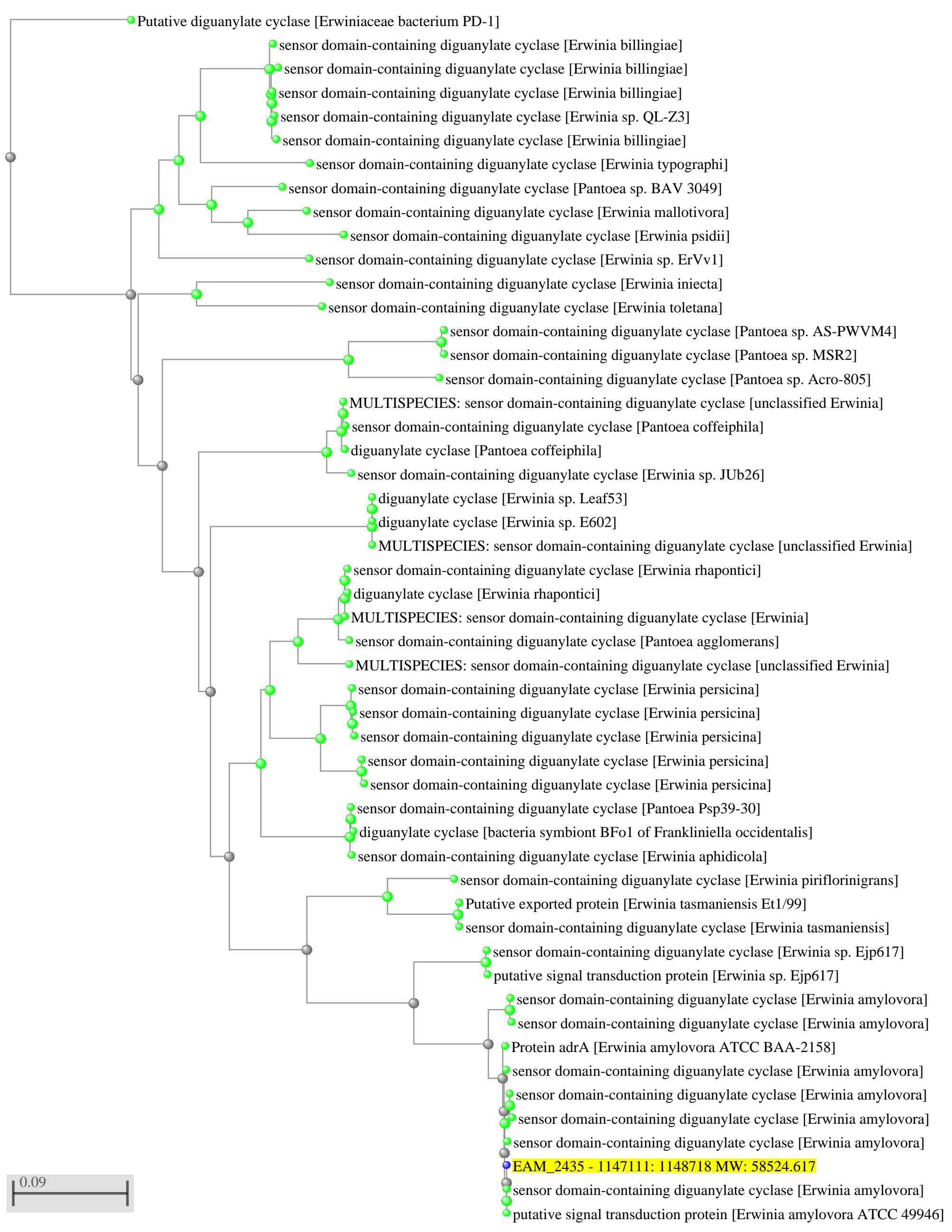

Supplement: S2 Datasheet — (PDF) [file ppat.1010737.s003.pdf]

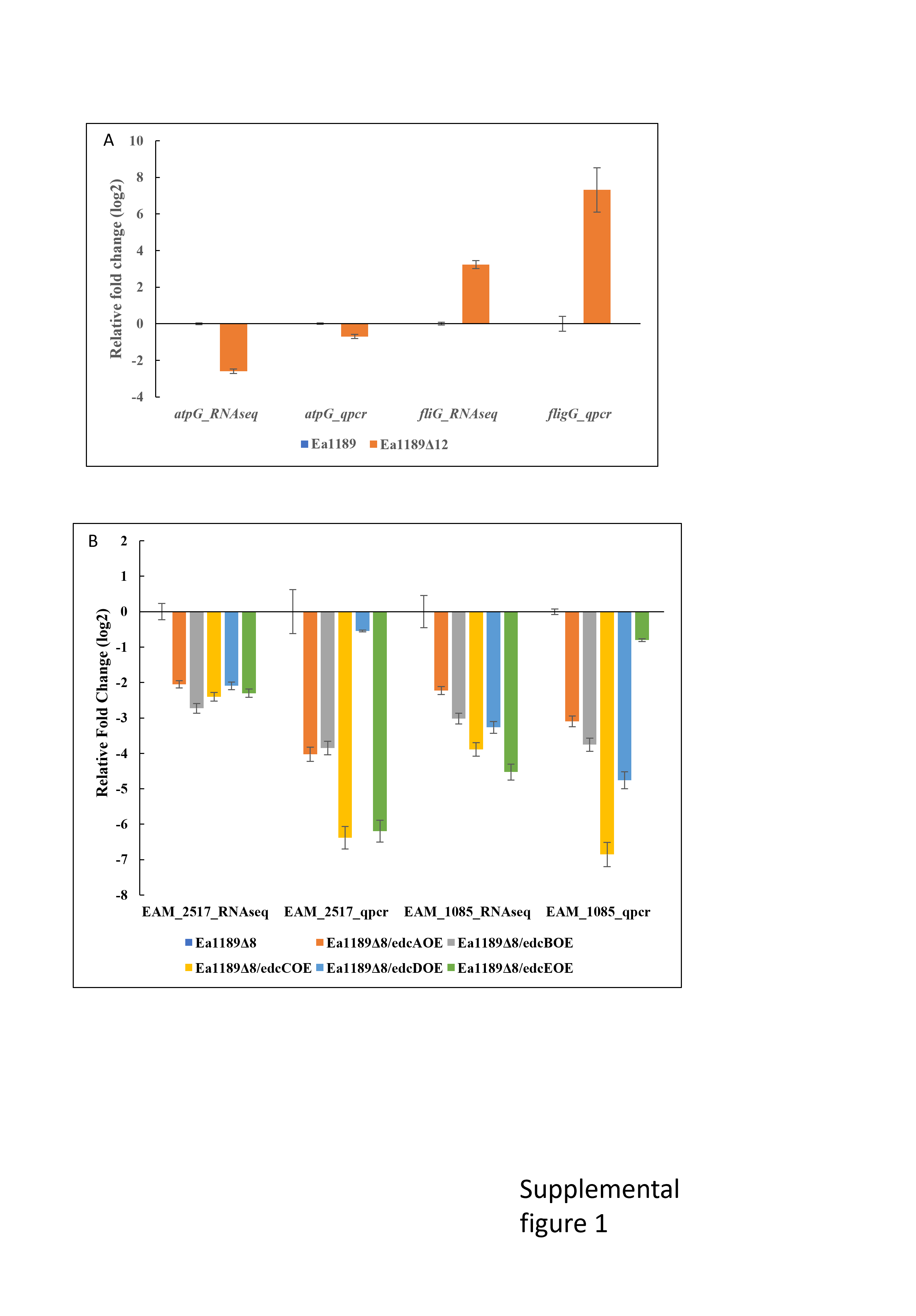

Supplement: S1 Fig — Graph summarizing the RNAseq and q-RT-PCR based examination of fold changes in the expression of representative genes (from both RNAseq studies) A) atpG and fliG in Ea1189Δ12 relative to WT Ea1189 and B) EAM_2517 and EAM_1085 in Ea1189Δ8 over expressing edcA-E individually relative to Ea1189Δ8. Error bars represent standard error of the means. (TIF) [file ppat.1010737.s004.tif]
